# Real-world effectiveness and safety of CDK4/6i in elderly and BIPOC patients with HR+/HER2- advanced/metastatic breast cancer: an updated systematic literature review

**DOI:** 10.3389/fonc.2025.1577075

**Published:** 2025-08-15

**Authors:** Nadia Harbeck, Adam Brufsky, Chloe Grace Rose, Beata Korytowsky, Connie Chen, Krista Tantakoun, Endri Jazexhi, Do Hoang Vien Nguyen, Meaghan Bartlett, Imtiaz A. Samjoo, Timothy Pluard

**Affiliations:** ^1^ Breast Center, Department of Gynecology & Obstetrics & Comprehensive Cancer Center Munich, Ludwig-Maximilians-University (LMU) University Hospital, Munich, Germany; ^2^ University of Pittsburgh Medical Center (UPMC) Hillman Cancer Center, University of Pittsburgh Medical Center, Pittsburgh, PA, United States; ^3^ Pfizer, Inc., New York, NY, United States; ^4^ Value & Evidence, EVERSANA™, Burlington, ON, Canada; ^5^ Hematology and Medical Oncology, St. Luke’s Cancer Institute, Kansas City, MO, United States

**Keywords:** CDK4/6i, breast, metastasis, real-world evidence, systematic literature review, elderly, BIPOC

## Abstract

**Background and aim:**

The HR-positive/HER2-negative (HR+/HER2-) advanced/metastatic breast cancer (a/mBC) treatment landscape has advanced with cyclin-dependent kinase 4/6 inhibitors (CDK4/6i), yet outcome disparities persist, particularly among older patients and black, indigenous, and people of color (BIPOC) communities. Emerging real-world evidence (RWE) since 2021 highlights the need for this updated systematic literature review.

**Methods:**

Searches were conducted in MEDLINE^®^, Embase^®^, and Cochrane Databases (07/06/2019–01/09/2024) and key congress proceedings (2020–2024). Studies on CDK4/6i treatment in elderly and BIPOC patients with ≥100 participants and details on therapy line and CDK4/6i type were included. Key outcomes for synthesis were effectiveness, treatment patterns, and safety.

**Results:**

This review included 23 unique studies. In comparisons of CDK4/6is among elderly patients, palbociclib and ribociclib demonstrated similar effectiveness, whereas data for abemaciclib were limited. These findings aligned with single-arm studies and CDK4/6i versus endocrine therapy (ET) comparisons, which demonstrated superior survival benefits for CDK4/6is over ET alone in both elderly and BIPOC subpopulations. Despite higher discontinuation rates and neutropenia in both subpopulations, survival outcomes remained unaffected in studies assessing effectiveness and tolerability.

**Conclusions:**

This review highlights that CDK4/6is are effective and well-tolerated in elderly and BIPOC patients with HR+/HER2− a/mBC. It also underscores the expanding body of RWE supporting CDK4/6is, highlighting their global use and key role in guiding clinical decisions, particularly for patient subpopulations underrepresented in clinical trials.

## Introduction

Breast cancer remains a significant global health concern, impacting diverse populations worldwide. According to the World Health Organization, breast cancer remains the most common cancer among women, with an estimated 2.3 million new cases diagnosed annually (1), and is the second leading cause of death in women, second only to lung cancer (2). In the United States (US), the American Cancer Society projects approximately 310,720 new cases of invasive breast cancer to be diagnosed in 2024 (3), emphasizing the persistent challenge this disease presents for the public. Among the various subtypes of breast cancer, hormone receptor (HR) positive (+), human epidermal growth factor receptor 2 negative (HER2-) breast cancer accounts for the majority (68%) of all cases (2). When this subtype progresses to advanced or metastatic stages (a/mBC), it presents unique challenges in terms of treatment and prognosis. Recent studies indicate that approximately 30% of patients initially diagnosed with early-stage HR+/HER2- breast cancer will eventually develop distant or metastatic disease (2), highlighting the critical need for effective treatment strategies for this patient population.

The landscape of HR+/HER2- a/mBC has seen significant advancements in recent years, particularly with the introduction of cyclin-dependent kinase 4/6 inhibitors (CDK4/6i) in 2015 (2) markedly improving survival outcomes when used in combination with endocrine therapy (ET) (4). However, despite these improvements, disparities in treatment outcomes persist, particularly among older patients (5) and black, indigenous, and people of color (BIPOC) communities (6). Recent findings highlight that while two-thirds of cancer patients are over 65 years old, only about 25% of cancer trial participants represent this age group (7). Similar disparities are also observed in BIPOC communities. Although overall breast cancer incidence rates are slightly lower in Black women compared to White women (8), Black women have a 40% higher mortality rate from breast cancer (9). Despite approximately 12.7% of the US population being Black with African or Caribbean ancestry, less than 3% of these patients are enrolled in clinical trials (6). This underrepresentation of high-risk subgroup populations in clinical studies results in critical gaps in clinical treatment guidelines, which are largely based on younger, non-BIPOC populations, who may exhibit different a/mBC disease characteristics and prognosis (5).

Newer treatment options for HR+/HER2- a/mBC have emerged in recent years with the approval of various CDK4/6i in the US. Since the approval of palbociclib (Ibrance^®^) by the US Food and Drug Administration (FDA) and European Medicines Agency in 2015 and 2016 respectively, for this indication (10, 11), followed by ribociclib (Kisqali^®^) in 2017 (12, 13) and abemaciclib (Verzenio^®^) in 2017 (14, 15), these agents have been widely adopted into clinical practice. While randomized controlled trials (RCTs) have been instrumental in establishing the efficacy and safety of CDK4/6i for treating HR+/HER2- a/mBC, they are limited by their restrictive inclusion and exclusion criteria (Brain et al., 2024). The controlled environment of these clinical trials may not fully represent the diverse patient populations affected by HR+/HER2- a/mBC, nor account for complexities and variation in real-world treatment patterns, adherence, and adverse events (AEs). Real-world evidence (RWE) can address these limitations by providing valuable insights into the effectiveness of CDK4/6i in HR+/HER2- a/mBC, particularly for patient subgroups that are frequently underrepresented in breast cancer RCTs, such as elderly and BIPOC populations. The RWE may also reveal emerging patterns of care over extended periods, especially after market approval.

A systematic literature review (SLR) evaluating RWE studies of CDK4/6i in the treatment of HR+/HER2- a/mBC, as well as an updated publication, has been previously published. Several years have passed since the original publication and the body of RWE for this class has grown considerably. As there is still a paucity of evidence summarizing CDK4/6i outcomes in elderly and BIPOC patients with HR+/HER2- a/mBC, the current study serves to help inform clinical decision making in these populations in real-world settings. Therefore, an updated SLR was conducted to synthesize and assess effectiveness, treatments patterns, and safety results in the patient subpopulations from RWE studies published since the previous review.

## Materials and methods

### Literature search

**Figure 1:**
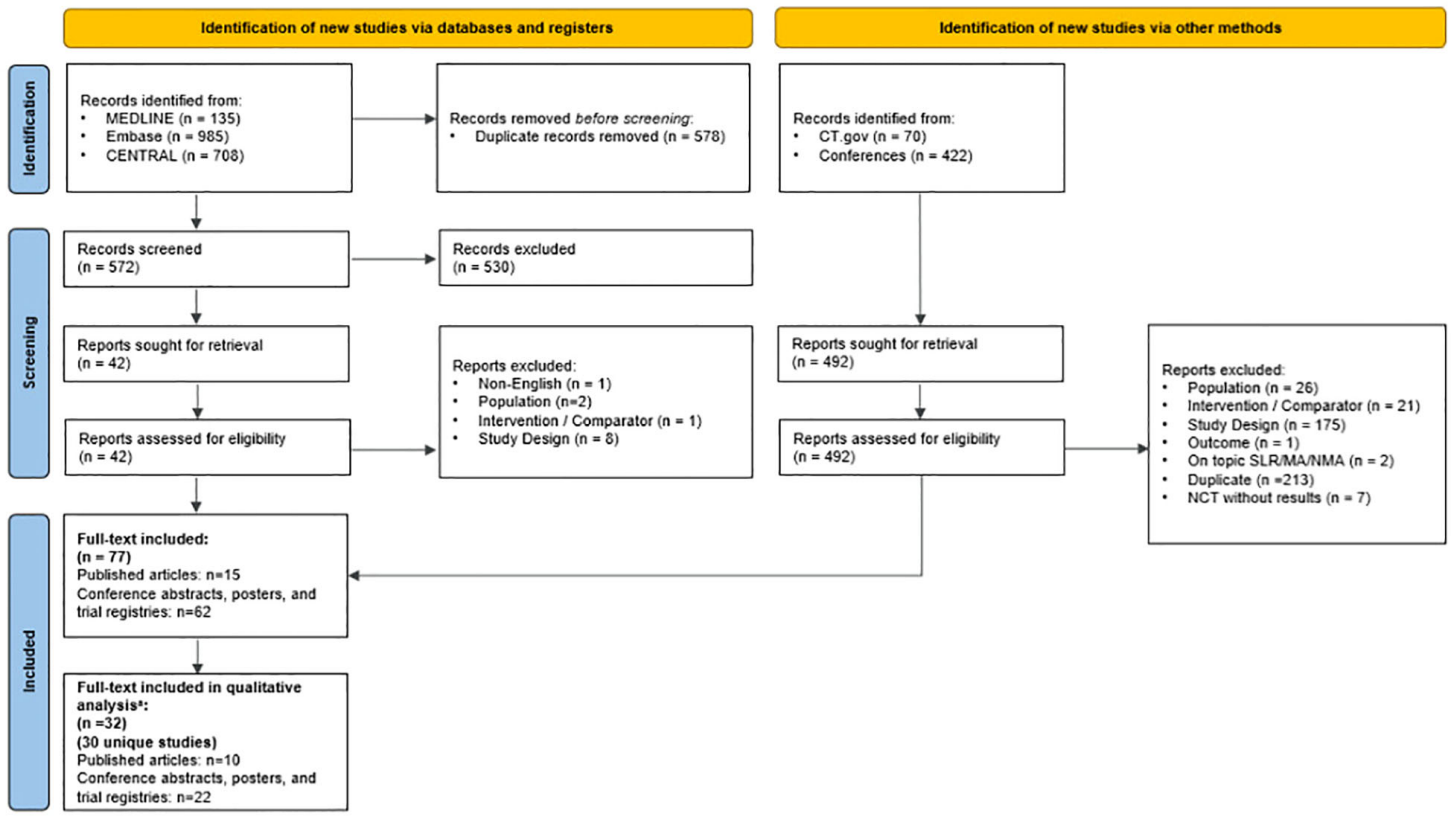
PRISMA diagram of the January 9, 2024 SLR Update. ^a^ Studies were excluded from the analysis if they had sample sizes less than 100 patients and did not specify the line of therapy or type of CDK4/6i assessed. MA, meta-analysis; NCT, National Clinical Trial; PRISMA, Preferred Reporting Items for Systematic Reviews and Meta-Analyses; SLR, systematic literature review. Source: The PRISMA 2020 statement: an updated guideline for reporting systematic reviews (16).

This SLR was conducted in accordance with the Preferred Reporting for Systematic Reviews and Meta Analyses (PRISMA) guidelines (16) (**Supplementary Materials**), as previously described (17). Updated searches were conducted and pooled to capture all data published since the previous SLR search on July 6, 2019 (17). These searches were performed on October 7, 2020; June 1, 2021; December 1, 2022; January 6, 2023; October 18, 2023; and January 9, 2024, using OVID Medline, EMBASE, the Cochrane Central Register of Controlled Trials, and the Cochrane Database of Systematic Reviews. Data from each search were collected in a consistent manner. The PRISMA diagram reflecting the most recent update on January 9, 2024 is shown in **Figure 1**. Diagrams from previous updates and details of the most recent search strategy can be found in the **Supplementary Materials**. This review was developed *a priori* for internal use only and was not registered.

Updated grey literature searches included bibliographies of relevant SLRs, ClinicalTrials.gov, and pre-specified key clinical conferences held between January 2022 and January 2024. These included the San Antonio Breast Cancer Symposium (SABCS), the American Society of Clinical Oncology (ASCO), the European Society for Medical Oncology (ESMO), ESMO BC, ESMO Asia, the International Society for Pharmacoeconomics and Outcomes Research (ISPOR), and ISPOR Europe (EU).

### Study selection and data extraction

Two independent reviewers assessed studies for eligibility using the systematic review software, DistillerSR (DistillerSR Inc., Ottawa, Ontario, Canada), following the previously described PICOS criteria (17). Any discrepancies between the reviewers during screening were resolved by consensus, with further disputes adjudicated by a third reviewer.

Studies were included if they reported RWE on patients aged ≥ 18 years with HR+/HER2- a/mBC receiving CDK4/6i treatment. Exclusion criteria included studies published in languages other than English or prior to 2019. For this analysis, only studies providing data on CDK4/6i treatment in elderly and BIPOC patients were considered. To enhance the robustness and relevance of the review findings, studies with fewer than 100 patients or those that did not specify the line of therapy or type of CDK4/6i were excluded. Outcomes of interest for the current synthesis include effectiveness outcomes (i.e., PFS and OS) with corresponding hazards ratios (HRs) (where available), treatment patterns (i.e., treatment discontinuation), and safety outcomes (i.e., neutropenia).

Data from the included publications were extracted into a standardized Microsoft^®^ Excel form (Microsoft Corporation, Seattle, US). Extraction was performed by a single reviewer and independently verified for accuracy and completeness by a second reviewer.

### Data analysis

Outcomes of interest were evaluated according to the type of CDK4/6i assessed (palbociclib, ribociclib, abemaciclib, or any CDK4/6i regimen), study design (single-arm or comparative), and patient population (older vs. younger or BIPOC vs. non-BIPOC). ‘Any CDK4/6i regimen’ referred to studies where a CDK4/6i—whether palbociclib, ribociclib, or abemaciclib—was evaluated, but results were not specific to an individual inhibitor. All data were summarized descriptively.

### Quality assessment

One independent reviewer evaluated all full-text publications for study quality using the Newcastle-Ottawa scale, which assesses cohort selection, comparability of cohorts, and assessment of outcomes in nonrandomized studies (18). Scores on the Newcastle-Ottawa scale range from 0 to 9: ≥ 7 indicated high-quality studies, 4−6 indicated moderate-quality studies, and < 4 indicated low-quality studies.

## Results

### Literature search & study selection

A total of 5,617 records were identified across the updated searches (4,043 from database searches and 1,574 from grey literature). After duplicates were removed, 4,837 records underwent screening at the title and abstract level, from which 2,491 full-text articles were retrieved for eligibility assessment. Ultimately, 882 records were deemed suitable for inclusion in the SLR. The search results, study selection procedures, and reasons for exclusion at the full-text stage for each update are detailed in the **Supplementary Materials**.

Of the 882 records included in the SLR, 860 were excluded from synthesis due to small sample sizes (fewer than 100 patients), unspecified line of therapy or type of CDK4/6i assessed, absence of outcomes of interest, or lack of specific data for elderly or BIPOC patients. Consequently, 23 unique studies (17 full-text articles and six conference abstracts/posters) reported effectiveness, treatment patterns, and/or safety data in elderly and/or BIPOC patients and were included in the qualitative analysis. A list of included studies is shown in the **Supplementary Materials**.

**Figure 2:**
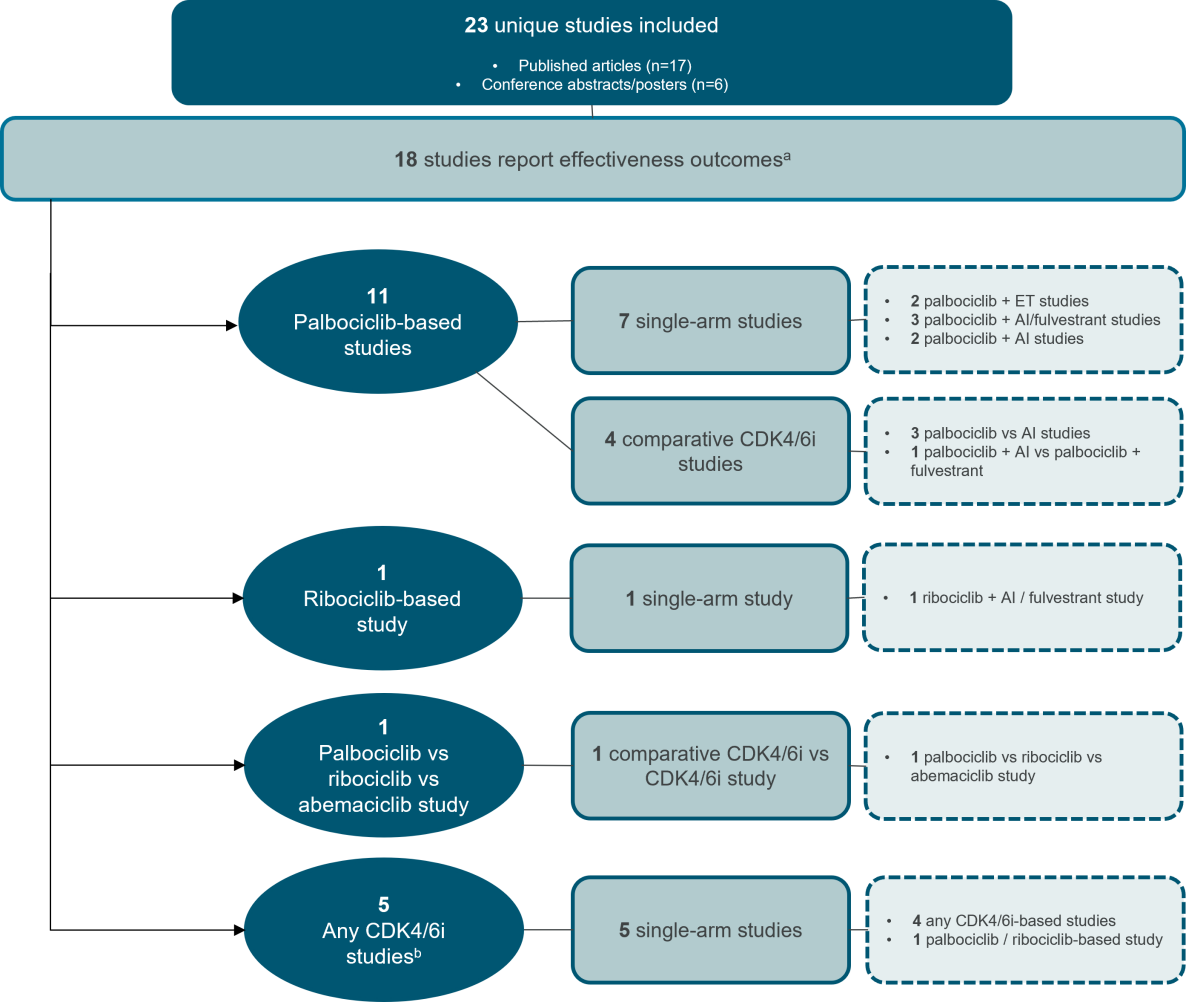
Study attrition diagram for studies that report effectiveness outcomes. ^a^Three studies reported both effectiveness and treatment patterns and/or safety outcomes (34–36), and were therefore included in both outcome categories. ^b^Any CDK4/6i regimen was defined as that in which a CDK4/6i—whether palbociclib, ribociclib, or abemaciclib—was evaluated, but the results were not specific to any individual CDK4/6i. AI, aromatase inhibitor, CDK4/6i, cyclin-dependent kinase 4/6 inhibitor; ET, endocrine therapy. Source: n=11 palbociclib-based studies (19–29) n=1 ribociclib-based study (30) n=1 palbociclib vs. ribociclib vs. abemaciclib study (31) n=5 any CDK4/6i studies (32–36).

**Figure 3:**
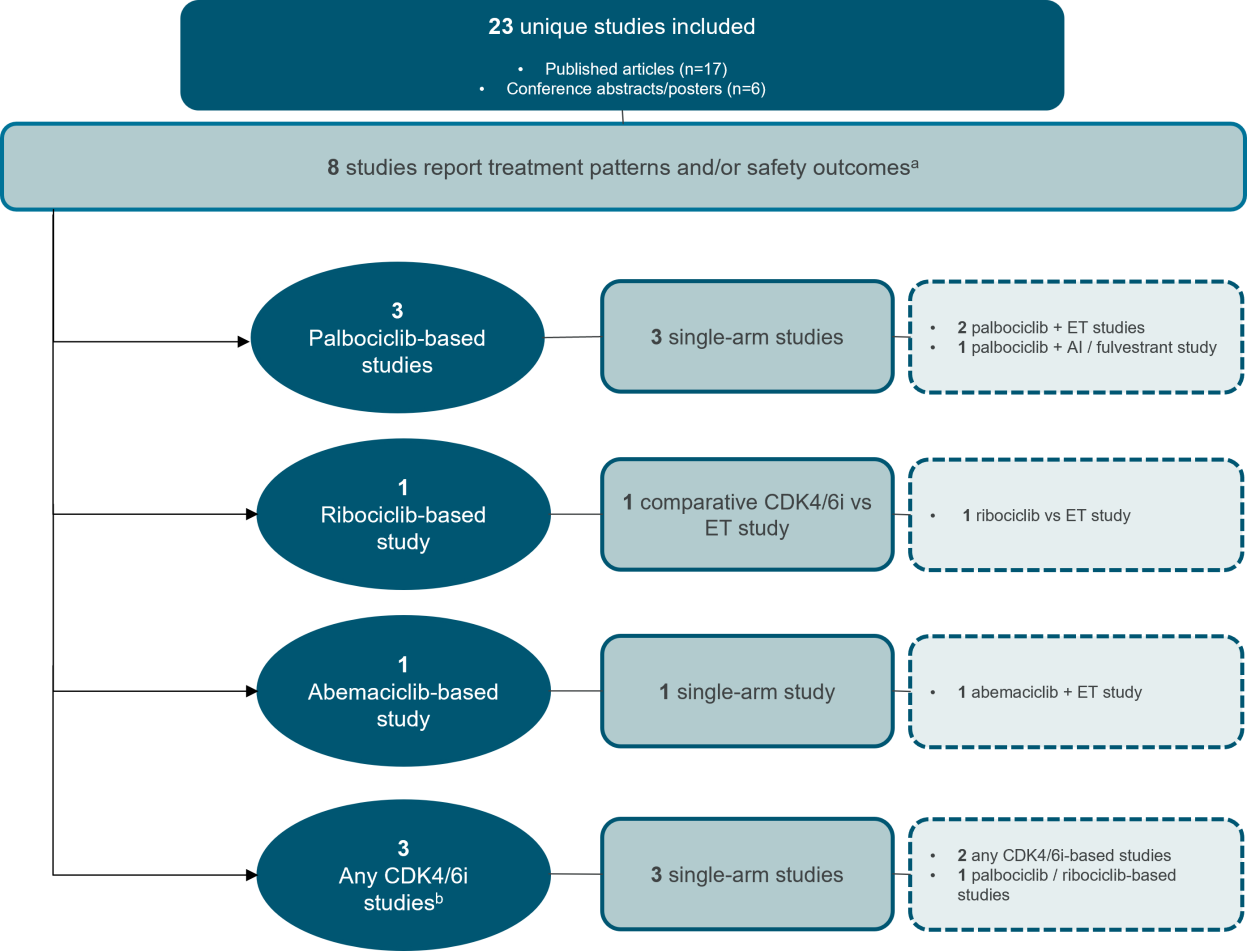
Study attrition diagram for studies that report treatment patterns and/or safety outcomes. ^a^Three studies reported both effectiveness and treatment patterns and/or safety outcomes (34–36), and were therefore included in both outcome categories. ^b^Any CDK4/6i regimen was defined as that in which a CDK4/6i—whether palbociclib, ribociclib, or abemaciclib—was evaluated, but the results were not specific to any individual CDK4/6i. AI, aromatase inhibitor; CDK4/6i, cyclin-dependent kinase 4/6 inhibitor; ET, endocrine therapy. Source: n=3 palbociclib-based studies (37–39) n=1 ribociclib-based study (40) n=1 abemaciclib-based study (41) n=3 any CDK4/6i studies (34–36).

Effectiveness outcomes were reported in most studies, with 18 providing PFS and/or OS data (**Figure 2**) (19–36). Treatment patterns and/or safety outcomes were reported in 8 studies (**Figure 3**) (34–41). Notably, three studies reported both effectiveness and treatment patterns and/or safety outcomes (34–36), and were therefore included in both outcome categories.

**Figure 4:**
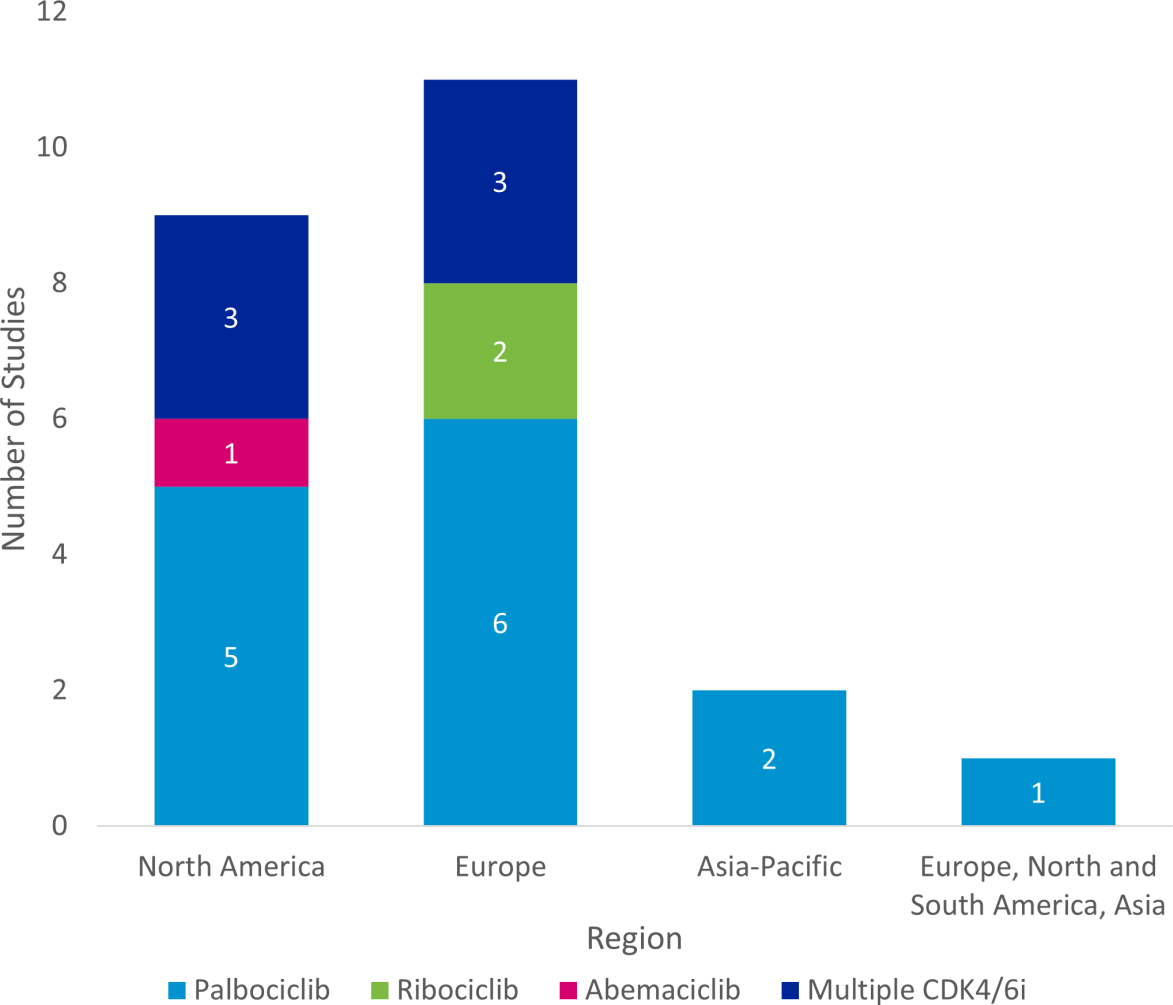
Regional distribution of included studies. ^a^ Studies were classified as “Multiple CDK4/6i” if two or more specified CDK4/6is were included in the study. CDK4/6i = cyclin-dependent kinase 4/6 inhibitor.

The majority of studies were conducted in Europe (n=11), followed by North America (n=9), and Asia-Pacific (n=2). One study was conducted across Europe, North and South America, and Asia (**Figure 4**).

### Quality assessment

The Newcastle-Ottawa assessment indicated that all full-text publications were of high or moderate quality (**Supplementary Materials**). Five studies received a score of 8 (26, 28, 29, 33, 41), demonstrating robust cohort selection, comparability, and outcome assessment, while one study scored 7 (31), indicating high quality despite minor limitations in comparability. Several studies scored between 5 and 6 (19, 21, 24, 25, 34–36), generally demonstrating adequate cohort selection but with limitations in comparability across cohorts and follow-up duration. Four studies scored 4 (20, 22, 23, 37), indicating moderate quality with notable methodological weaknesses.

### Effectiveness of CDK4/6i in RWE studies

Of the 18 studies that reporting either PFS (n=9), OS (n=1), or both (n=8), 11 focused on palbociclib (19–29), one on ribociclib (30), and one directly compared different CDK4/6is (31). The other five studies evaluated CDK4/6i regimens collectively, without specifying results for individual inhibitors (32–36). No studies assessing abemaciclib reported effectiveness outcomes of interest.

#### Palbociclib

Effectiveness data for elderly and/or BIPOC patients receiving palbociclib were reported in seven single-arm studies (19, 21–25, 27) and four comparative studies evaluating palbociclib versus ET (**Figure 2**, **Tables 1**, **2**) (26, 28, 29). Of these 11 studies, four were from the US (19, 26, 28, 29), three were from the European Union (EU) (21, 23, 27), two were from China (22, 25), one was from the United Kingdom (24), and one was from 13 countries spanning Europe, North and South American, and Asia (20). The age range of elderly patients spanned from <50 (19, 24) to ≥75 (19). BIPOC data was reported for Black or African American (19, 20, 29), Hispanic, Middle Eastern, Asian, or ‘Other’ (including Native American, mixed race, and not specified) patients (20).

**Table 1:** Effectiveness outcomes for elderly patients receiving CDK4/6i in RWE studies.

Study name; reference	Country	Treatment (line of therapy)	Age (years)	Sample size	PFS			OS		
					Median (95% CI), months	HR (95% CI); *P* value	At latest timepoint, n (%)	Median (95% CI), months	HR (95% CI); *P* value	At latest timepoint, n (%)
*Palbociclib Studies*										
PALBOSPAIN; 4203- Anton-2023	Spain	Palbo + ET (1L)	<50	130	27 (21 – 35)	NR	NR	NR	NR	NR
			50-70	376	21 (19 – 26)	NR	NR	NR	NR	NR
			>70	219	27 (22 – 37)	NR	NR	NR	NR	NR
3309-Wu-2023	China	Palbo + ET (All lines)	<65	293	14.23 (NR)	NR	NR	NR	NR	NR
			≥65	104	14.17 (NR)	NR	NR	NR	NR	NR
1804-Ismail-2021	Netherlands	Palbo + AI or Ful (All lines)	<70	409	NR	NR	NR	26.7 (23.5 – 29.7)	NR	NR
			≥70	189	NR	NR	NR	20.7 (18.4 – 31.5)	NR	NR
1624-Zhang-2021	China	Palbo + AI or Ful (1L to 3L)	<55	65	11.5 (2.31 – 20.7)	NR	NR	NR	NR	NR
			≥55	86	12.8 (9.44 – 16.16)	NR	NR	NR	NR	NR
1773-Sampedro-2021	Spain	Palbo + Let, Exe, or Ful (All lines)	<65	38	15 (9.3 – 20.7)	NR	NR	NR	NR	NR
			≥65	35	24 (11.8 – 36.2)	NR	NR	NR	NR	NR
1809-El Badri-2021	UK	Palbo + AI (1L)	Elderly	276	NR	NR	24 months: 179 (64.9)	NR	NR	24 months: 204 (74)
366-Law-2022	US	Palbo + AI (1L)	<50^a^	25	NR (13.3 – N/R)	NR	NR	NR	NR	NR
			50-64^a^	86	26.5 (17.4 – N/R)	NR	NR	NR	NR	NR
			65-74^a^	80	41.9 (29.8 – N/R)	NR	NR	NR	NR	NR
			≥75^a^	51	35.8 (21.2 – N/R)	NR	NR	NR	NR	NR
IRIS; 261-Mycock-2021	Europe, North and South America, and Asia^b^	Palbo + AI (All lines)	<65	917 PFS/910 OS	NR	NR	24 months: NR (61.6)	NR	NR	24 months: NR (92.8)
			65+	829/825	NR	NR	24 months: NR (70.5)	NR	NR	24 months: NR (87.4)
		Palbo + Ful (All lines)	<65	622/607	NR	NR	24 months: NR (52.3)	NR	NR	24 months: NR (88.9)
			65+	584/565	NR	NR	24 months: NR (49.3)	NR	NR	24 months: NR (86.6)
Rugo 2023b; 3494-Rugo-2023	US	Palbo + Let (1L)	Elderly (sIPTW)	450	22.2 (12.9–18.9)	0.59 (0.47-0.74); *P*<0.001	NR	N/R	0.55 (0.42-0.72); *P*<0.001	NR
		Let (1L)	Elderly (sIPTW)	335	15.8 (20.0 – 30.4)		NR	43.4 (30.0 – NE)		NR
P-REALITY-X; 4379-Brufsky-2023	US	Palbo + AI (1L)	Elderly (sIPTW)	371	20 (15.7 – 26.7)	0.72 (0.59-0.89); *P*=0.0021	NR	43 (40.1 – NE)	0.66 (0.51-0.84); *P*=0.0007	NR
		AI (1L)	Elderly (sIPTW)	287	15 (12.9 – 16.8)		NR	32.4 (28.2 – 38.2)		NR
*Ribociclib studies*										
REACHAUT; SABCS23-117-Singer-2023	Austria	Ribo + AI/Ful (1L)	<75	221	28.7 (22.9 – 56.6)	NR	NR	NR	NR	NR
			≥75	60	29.7 (23.2 – 46.0)	NR	NR	NR	NR	NR
*Palbociclib vs. ribociclib vs. abemaciclib*										
4506-Tang-2023	UK	CDK4/6i + ET (1L)	≤65	97	28 (NR)	NR	5 years: NR (22.79)	77.4 (NR)	NR	5 years: NR (52)
			66-79	95	29 (NR)	NR	5 years: NR (27.73)	61.7 (NR)	NR	5 years: NR (51.16)
			≥80	35	21.3 (NR)	NR	5 years: NR (11.89)	35 (NR)	NR	5 years: NR (32.28)
		Palbo + ET (1L)	≤65	62	30.2 (NR)	NR	5 years: NR (25.56)	77.4 (NR)	NR	5 years: NR (55.27)
			66-79	75	28.2 (NR)	NR	5 years: NR (25.61)	61.7 (NR)	NR	5 years: NR (50.17)
			≥80	25	14.5 (NR)	NR	5 years: NR (0)	29.6 (NR)	NR	5 years: NR (23.34)
		Ribo + ET (1L)	≤65	25	20.5 (NR)	NR	5 years: NR (27.93)	44.6 (NR)	NR	5 years: NR (36.86)
			66-79	16	24.7 (NR)	NR	5 years: NR (34.29)	54.8 (NR)	NR	5 years: NR (49.36)
			≥80	5	68.2 (NR)	NR	5 years: NR (60)	NR (NR)	NR	5 years: NR (68.86)
		Abema + ET (1L)	≤65	8	NR	NR	5 years: NR	36.5 (NR)	NR	5 years: NR
			66-79	6	NR	NR	5 years: NR	NR	NR	5 years: NR
			≥80	5	NR	NR	5 years: NR	NR	NR	5 years: NR

a Age at diagnosis.

b Countries include Argentina, Germany, Canada, Belgium, Italy, Spain, Switzerland, Netherlands, Japan, France, Portugal, US, and the UK.

1L, first-line; 3L, third-line; Abema, abemaciclib; AI, aromatase inhibitor; CDK4/6i, cyclin-dependent kinase 4/6 inhibitor; CI, confidence interval; ET, endocrine therapy; Exe, exemestane; Ful, fulvestrant; Let, letrozole; N/R, not reached; NR, not reported; OS, overall survival; Palbo, palbociclib; PFS, progression-free survival; Ribo, ribociclib; RWE, real-world evidence; UK, United Kingdom; US, United States

**Table 2:** Effectiveness outcomes for BIPOC patients receiving CDK4/6i in RWE studies.

Study name; reference	Country	Treatment (line of therapy)	Race/ethnicity subgroup	Sample size	PFS			OS			
					Median (95% CI), months	HR (95% CI); *P* value	At latest timepoint, n (%)	Median (95% CI), months		HR (95% CI); *P* value	At latest timepoint, n (%)
366-Law-2022	US	Palbo + AI (1L)	White	196	35.8 (24.4 – N/R)	NR	NR	NR		NR	NR
			Black or African American	29	18.5 (13.8 – N/R)	NR	NR	NR		NR	NR
IRIS; 261-Mycock-2021	Europe, North and South America, and Asia^a^	Palbo + AI (All lines)	White (non-Hispanic)	1258 PFS/1251 OS	NR	NR	24 months: NR (89.0)	NR		NR	24 months: NR (92.1)
			Black (non-Hispanic)	99/96	NR	NR	24 months: NR (81.8)	NR		NR	24 months: NR (93.2)
			Hispanic	132	NR	NR	24 months: NR (81.3)	NR		NR	24 months: NR (72.4)
			Middle Eastern	42	NR	NR	24 months: NR (90.9)	NR		NR	24 months: NR (91.7)
			Asian	159/158	NR	NR	24 months: NR (44.6)	NR		NR	24 months: NR (88.0)
			Other (Native American, mixed race, not specified)	56	NR	NR	24 months: NR (87.0)	NR		NR	24 months: NR (94.6)
		Palbo + Ful (All lines)	White (non-Hispanic)	862/842	NR	NR	24 months: NR (45.3)	NR		NR	24 months: NR (86.5)
			Black (non-Hispanic)	57	NR	NR	24 months: NR (92.1)	NR		NR	24 months: NR (93.4)
			Hispanic	82/79	NR	NR	24 months: NR (46.0)	NR		NR	24 months: NR (95.2)
			Middle Eastern	35/32	NR	NR	24 months: NR (48.0)	NR		NR	24 months: NR (96.0)
			Asian	123/119	NR	NR	24 months: NR (69.6)	NR		NR	24 months: NR (91.9)
			Other (Native American, mixed race, not specified)	47/43	NR	NR	24 months: NR (79.1)	NR		NR	24 months: NR (90.0)
Rugo 2023a; 4549-Rugo-2023	US	Palbo + AI (1L)	African-American	127	18 (12.4 – 26.7)	0.72 (0.48 – 1.07); *P* = 0.102	20 months: NR (46.6)	N/R (38.2 – NR)		0.54 (0.35 – 0.84); *P* = 0.007	36 months: NR (61.2)
		AI (1L)	African-American	143	10.5 (7.0 – 13.4)		20 months: NR (30.1)	28.2 (19.2 – 52.8)			36 months: NR (44.3)

a Countries include Argentina, Germany, Canada, Belgium, Italy, Spain, Switzerland, Netherlands, Japan, France, Portugal, US, and the UK.

1L, first-line; AI, aromatase inhibitor; CDK4/6i, cyclin-dependent kinase 4/6 inhibitor; CI, confidence interval; Ful, fulvestrant; N/R, not reached; NR, not reported; OS, overall survival; Palbo, palbociclib; PFS, progression-free survival; RWE, real-world evidence; US, United States

In two single-arm studies evaluating palbociclib plus ET, median PFS was similar between older and younger patients in both the first-line and all-lines settings (25, 27). Also, in one study they noted median PFS was consistently higher in the first-line setting, ranging from 21 (n=376 [50–70 subgroup]) to 27 months (n=130 [<50 subgroup]; n=219 [>70 subgroup]) (27), compared to 14.2 months (n=104 [≥65 subgroup]; n=293 [<65 subgroup]) when all lines of therapy were considered (25). These findings are consistent with observations made in the 2024 SLR by Brain and colleagues, which found PFS was comparable between the older and younger patient cohorts (Brain et al., 2024). Palbociclib in combination with AI or fulvestrant was evaluated in three single-arm studies, two of which used descriptive statistics (21, 23) and one that used multivariate analysis for PFS (22). In two of these, median PFS was higher in older patients compared to younger patients, regardless of the line of therapy (21, 22). However, in the third study, median OS was lower in patients ≥70 years compared to those who were younger (26.7 months [n=409] vs. 20.7 months [n=189], respectively) (23). First-line palbociclib plus AI was evaluated in two single-arm studies (19, 24). Descriptive analyses from one US-based study compared outcomes between older (65–74 and ≥75 years) and younger patients (<50 and 50–64 years), finding that median PFS was consistently higher in older patients, with the greatest benefit observed in those aged 65–74 at diagnosis (19). The same study also reported that median PFS was notably lower among Black or African American patients compared to White patients (18.5 months [n=29] vs. 35.8 months [n=196], respectively) (19). However, these results should be interpreted with caution due to the small sample size in the Black or African American patient cohort. Multivariable cox regression results from the second study, which only reported outcomes for elderly patients, found a 24-month PFS rate of 64.9% and a 24-month OS rate of 74% (n=276) (42). Of the six full-text studies with quality assessments, all were judged to be of moderate quality (three had NOS scores of 4 (19, 22, 23), two had NOS scores of 5 (19, 24, 25), and one had a NOS score of 6 (21).

In three US-based comparative studies evaluating palbociclib versus ET, both PFS and OS were consistently improved in elderly and BIPOC subpopulations treated with palbociclib plus AI compared to those receiving AI alone in the first-line setting (26, 28, 29). All studies leveraged sIPTW to adjust for patient demographics and clinical characteristics. For elderly patients, palbociclib plus AI demonstrated a median PFS ranging from 20 (n=371) to 22.2 months (n=450) and a median OS of 43 months (n=371) (not reached in one study after a median follow-up of 20.2 months; n=450). In contrast, AI alone had a median PFS of 15 (n=287) to 15.8 months (n=335) and an OS ranging from 32.4 (n=287) to 43.4 months (n=335) (26, 28). In both comparative studies, PFS and OS were significantly improved with palbociclib (plus letrozole in Rugo 2023b; plus AI in Brufsky 2023 [P-REALITY-X]) in the first-line setting compared with the control treatment (letrozole in Rugo 2023b; AI in Brufsky 2023 [P-REALITY-X]). Similarly, in African American patients, first-line treatment with palbociclib plus AI showed improved effectiveness outcomes, with a median PFS of 18 months and a median OS not reached (median follow-up of 24.0 months; n=127), compared to AI alone, which had a median PFS of 10.5 months and a median OS of 28.2 months (median follow-up of 18.2 months; n=143) (29). In this comparative study, there was no significant improvement in PFS, but OS was significantly improved with palbociclib plus AI compared with AI alone. Palbociclib, in combination with AI or fulvestrant, was evaluated in an international comparative study (20). The IRIS study reported comparable 24-month PFS rates between younger and older patients when all lines of therapy were considered; however, no test for significance was conducted (palbociclib+AI: 61.6% [<65 subgroup] vs. 70.5% [≥65 subgroup]; palbociblib+fulvestrant: 52.3% [<65 subgroup] vs. 49.3% [≥65 subgroup]). Similar results were observed for 24-month OS rates. This study also reported 24-month PFS and OS rates for BIPOC patients. Following treatment with palbociclib+AI, PFS rates were lowest among Asian patients and highest in the Middle Eastern patient group (44.6% vs. 90.9%, respectively). However, the OS rates in this treatment group were lowest among Hispanic patients and highest for the ‘Other’ category (72.4% vs. 94.6%, respectively; includes Native American, mixed race, and not specified). Within the palbociclib+fulvestrant treatment group, PFS rates were lowest for White patients (45.3%), and Hispanic patients (46.0%), and highest among Black patients (92.1%). Similarly, OS rates were lowest within the White patient subgroup (86.5%), followed by ‘Other’ patients (90.0%), but highest among Middle Eastern patients (96.0%). All four studies underwent quality assessment; three were identified as being high-quality (NOS scores of 8) (26, 28, 29) and one was identified as moderate-quality (NOS score of 4) (20).

#### Ribociclib

An EU conference abstract of the single-arm REACHAUT study compared effectiveness data for elderly patients <75 years and ≥75 years (30). Descriptive analyses demonstrated comparable median PFS between patients aged ≥75 years (29.7 months; n=160) and younger patients (28.7 months; n=221) receiving ribociclib plus AI or fulvestrant in the first-line setting, after a median follow-up of 14.4 months (**Figure 2**, **Table 1**) (30).

No studies evaluating ribociclib in BIPOC subpopulations reported PFS or OS data.

#### Palbociclib vs. Ribociclib vs. Abemaciclib

A single comparative study conducted in the United Kingdom by Tang et al., with a median follow-up of 49 months, directly compared first-line palbociclib, ribociclib, and abemaciclib among different age groups: ≤65 years, 66–79 years, and ≥80 years (**Figure 2**, **Table 1**) (31). No test for significance for PFS or OS were performed in any age group. Additional multivariate analysis was conducted for OS to account for the following factors: *de novo* disease, PR status, age, ER scores, histopathology, and CDK4/6i.

The authors highlighted that despite having an older patient cohort (median age 69 years) compared to those in the MONALEESA-2 and PALOMA-2 studies (median age 62 years), median PFS and OS were comparable between palbociclib (n=162) and ribociclib (n=46), with no significant differences observed (median PFS: 27.5 months vs. 25.7 months, p = 0.3; median OS: 49.5 months vs. 50.2 months, p = 0.67).

In Tang et al., the results further revealed that palbociclib provided the longest PFS in patients aged ≤65 years, with a median of 30.2 months, and an OS of 77.4 months (n=62), whereas ribociclib had a PFS of 20.5 months and an OS of 44.6 months (n=25). Abemaciclib showed a PFS that was not reached and an OS of 36.5 months (n=8) (31). For patients aged 66–79 years, palbociclib maintained favorable outcomes, though slightly lower relative to the younger palbociclib group, with a PFS of 28.2 months and an OS of 61.7 months (n=75). Ribociclib, however, demonstrated improved results in this age group compared to ribociclib younger patients, with a PFS of 24.7 months and an OS of 54.8 months (n=16) (31). In patients aged ≥80 years, outcomes were more variable across treatments however there were very few patients in this group. Palbociclib showed a marked reduction in both PFS and OS compared to younger age groups, with a PFS of 14.5 months and an OS of 29.6 months (n=25). In contrast, ribociclib showed significantly better outcomes in this elderly group, achieving a PFS of 68.2 months and a 5-year OS rate of 68.86% (n=5) (31). However, results should be interpreted with caution, as most subgroups had small samples sizes (<30).

The multivariate analysis in Tang et al. revealed *de novo* disease (p = 0.0007) and age (≤65 years; p = 0.0225) were significantly independently associated with prolonged median PFS and median OS. In particular, when examining palbociclib between younger and older patients, there was a decline in efficacy with age, with younger patients showing better survival outcomes. Conversely, ribociclib demonstrated improved outcomes in older patients compared to their younger counterparts (31). Limited data for abemaciclib in older age groups precluded a meaningful comparison with palbociclib and ribociclib. This study was assessed as high-quality, with a NOS score of 7 (31).

No studies comparing CDK4/6is head-to-head in BIPOC subpopulations reported PFS or OS data.

#### Effectiveness of any CDK4/6i regimen in RWE studies

Five single-arm studies also reported effectiveness outcomes for any CDK4/6i regimens used in elderly or BIPOC subgroups (**Figure 2**, **Tables 3**, **4**) (32–36).

**Table 3:** Effectiveness outcomes for elderly patients receiving any CDK4/6i in RWE studies.

Study name; reference	Country	Treatment (line of therapy)	Subgroup	Sample size	PFS			OS		
					Median (95% CI), months	HR (95% CI); *P* value	At latest timepoint, n (%)	Median (95% CI), months	HR (95% CI); *P* value	At latest timepoint, n (%)
204-Pla-2022	Spain	CDK4/6i (1L)	Elderly	NR	27.2 (11.2 – N/R)	NR	NR	NR	NR	NR
		CDK4/6i (2L)	Elderly	NR	4.1 (3.1 – N/R)	NR	NR	NR	NR	NR
		CDK4/6i (3L+)	Elderly	NR	10.5 (7.3 – N/R)	NR	NR	NR	NR	NR
Olazagasti 2023; 3585- Olazagasti-2023	US	CDK4/6i (All lines)	< 70 years	129	21.9 (16.9–34.2)	NR	NR	NR	NR	NR
			≥70 years	73	18.2 (11.7 – N/R)	NR	NR	NR	NR	NR
606- Fountzilas-2020	Greece	Palbo or ribo + ET (All lines)	>75 years old	43	10.9 (4.5 – N/R)	NR	NR	24.2 (19.9 – N/R)	NR	NR
		Palbo or ribo + ET (1L)	>75 years old	20	10.9 (3.1 – 24.2)	NR	NR	24.2 (10.9 – 24.2)	NR	NR
		Palbo or ribo + ET (2L+)	>75 years old	23	7.5 (4.5 – N/R)	NR	NR	N/R	NR	NR

1L, first-line; 2L, second-line; 3L, third-line; CDK4/6i, cyclin-dependent kinase 4/6 inhibitor; CI, confidence interval; ET, endocrine therapy; N/R, not reached; NR, not reported; OS, overall survival; Palbo, palbociclib; PFS, progression-free survival; Ribo, ribociclib; RWE, real-world evidence; US, United States.

**Table 4:** Effectiveness outcomes for BIPOC patients receiving any CDK4/6i in RWE studies.

Study name; reference	Country	Treatment (line of therapy)	Subgroup	Sample size	PFS			OS			
					Median (95% CI), months	HR (95% CI); *P* value	At latest timepoint, n (%)	Median (95% CI), months		HR (95% CI); *P* value	At latest timepoint, n (%)
271-Mouabbi-2022	US	CDK4/6i + ET (All lines)	IDC, Black	165	NR	NR	11.7 (NR)	NR		NR	28.1 (NR)
			ILC, Black	17	NR	NR	7.5 (NR)	NR		NR	16.0 (NR)
1198-Schreier-2022	US	CDK4/6i + Let, Ful, Ana, Exeor Tam (All lines)	Black patients	83	26.3 (17.1 – 42.7)	NR	NR	NR		NR	NR
			Non-Black patients	99	33.9 (27.1 – 49.8)	NR	NR	NR		NR	NR
		CDK4/6i + Let, Ful, Ana, Exe or Tam (1L)	Black patients	NR	32.5 (26.0 – N/R)	NR	NR	NR		NR	NR
			Black patients with neutropenia	NR	32.5 (26.0 – N/R)	NR	NR	NR		NR	NR
			Black patients with dose reduction	NR	26.3 (18.6 – N/R)	NR	NR	NR		NR	NR
			Non-Black patients	NR	43.2 (33.4 – N/R)	NR	NR	NR		NR	NR
			Non-Black patients with neutropenia	NR	43.2 (33.4 – N/R)	NR	NR	NR		NR	NR
			Non-Black patients with dose reduction	NR	52.3 (33.9 – N/R)	NR	NR	NR		NR	NR

1L, first-line; Ana, anastrozole; CDK4/6i, cyclin-dependent kinase 4/6 inhibitor; CI, confidence interval; ET, endocrine therapy; Exe, exemestane; Ful, fulvestrant; IDC, invasive duct carcinoma; ILC, invasive lobular carcinoma; Let, letrozole; N/R, not reached; NR, not reported; OS, overall survival; PFS, progression-free survival; RWE, real-world evidence; Tam, tamoxifen; US, United States.

Two studies evaluated CDK4/6i plus ET in Black patients (33, 35), though only one compared outcomes to non-Black patients. In this US study, Black patients had consistently shorter median PFS across all lines of therapy compared to non-Black patients (35). Additionally, median PFS was similar in patients with neutropenia, whereas those with dose reductions demonstrated longer PFS (35). The second study compared Black patients with invasive lobular carcinoma (ILC) to those with invasive duct carcinoma (IDC) and found that IDC patients had higher survival rates (33). No test for significance was conducted for PFS or OS in either study. Quality assessment was performed for both studies, with one judged to be high-quality (NOS score of 8) and the other moderate-quality (NOS score of 5).

Two studies evaluated CDK4/6i monotherapy in elderly patients (32, 36), however, only one made comparisons to younger patients. In this US-based study, median PFS was shorter in patients age ≥70 compared to younger patients (18.2 [n=73] vs. 21.9 months [n=129], respectively) (36). The other study assessed PFS by line of therapy, showing the greatest improvement in first-line treatment, followed by third-line or later, with second-line therapy yielding the least improvement (32). However, it should be noted that these results were derived from a conference abstract from Spain, in which the sample sizes were not reported (32). Lastly, a study examining palbociclib or ribociclib plus ET in patients >75 years found similar PFS and OS outcomes between first-line and all-lines treatment settings (34). No test for significance was conducted for PFS or OS. Quality assessment was performed for two of the three studies, with both receiving NOS scores of 5 and being considered of moderate-quality.

### Treatment patterns and safety outcomes of CDK4/6i in RWE studies

Of the eight studies reporting either treatment patterns (n=3), safety outcomes (n=3), or both (n=2), three focused on palbociclib (37–39), one on ribociclib (40), and one on abemaciclib (41). Notably, none of these studies reported relevant data in BIPOC subpopulations. The remaining three studies analyzed CDK4/6i regimens collectively, without specifying results for individual inhibitors (34–36). Four of the eight studies were conducted in the US and four were conducted in the EU, with the age of elderly patients ranging from 18 years to >85 years.

#### Palbociclib

Three descriptive single-arm studies reported treatment discontinuations and/or neutropenia data for elderly patients receiving palbociclib (**Figure 3**, **Table 5**) (37–39). Of these, two evaluated palbociclib plus ET in the first-line setting (38, 39). In a conference abstract of the PERFORM study, the rate of treatment discontinuation for any reason was similar between older (≥75 years) and younger patients. However, the discontinuation rate due to AEs was higher in older patients (9.2% [n=185] vs. 3.0% [n=439], respectively) (39). The PalomAGE study abstract, was a study focusing entirely on elderly patients (≥70 years), reported a treatment discontinuation rate of 37.8% for any reason and 7.7% specifically due to adverse events with palbociclib plus ET (n=362). Additionally, neutropenia occurred in 54.4% of patients overall, with 41.1% experiencing grade 3/4 severity (38). In the third study, patient age (<60 years vs. ≥60 years of age) was not a significant contributing factor to observed rates of dose reductions or neutropenia-related treatment discontinuation with palbociclib plus AI or fulvestrant. This study was assessed as moderate-quality, with a NOS score of 4 (37).

**Table 5:** Treatment patterns and safety outcomes for elderly patients receiving CDK4/6i in RWE studies.

Study name; reference	Treatment (Line of therapy)	Subgroup	Sample size	Treatment discontinuation, n (%)	Treatment discontinuation due to AEs, n (%)	Overall neutropenia, n (%)	Grade 3/4 neutropenia, n (%)
*Palbociclib Studies*							
PalomAGE; ASCO23-011-Carola-2023	Palbo + ET (1L)	≥70 years (Cohort A)	362	137 (37.8)	NR (7.7)	196 (54.4)	148 (41.1)
PERFORM; ESMO23-012-Radosa-2023	Palbociclib + ET (1L)	< 75 years of age	439	120 (27.3)	Serious AEs: 13 (3)	NR	NR
		≥ 75 years of age	185	53 (28.6)	Serious AEs: 17 (9.2)	NR	NR
1973-Dennison-2021	Palbo + AI or Ful (All lines)	<60 years old	48	48 (100)	NR	NR	NR
		≥60 years old	59	39 (100)	NR	NR	NR
*Ribociclib Studies*							
RIBANNA; ESMO23-052-Decker-2023	Ribo + ET (1L)	75–80 years of age	281	NR	NR	75 (23.66)	46 (14.51)
		>80 years of age	153	NR	NR	35 (20)	15 (8.57)
	ET (1L)	75–80 years of age	42	NR	NR	2 (4.26)	2 (4.26)
		>80 years of age	41	NR	NR	1 (2.08)	0 (0)
*Abemaciclib Studies*							
3517-Ring-2023	Abema + ET (All lines)	Age 18-49	42	NR	NR (19)	NR	NR
		Age 50-64	155	NR	NR (25.2)	NR	NR
		Age 65-74	138	NR	NR (31.9)	NR	NR
		Age 75-84	82	NR	NR (36.6)	NR	NR
		Age 85+	31	NR	NR (48.4)	NR	NR

1L, first-line; Abema, abemaciclib; AI, aromatase inhibitor; AE, adverse event; CDK4/6i, cyclin-dependent kinase 4/6 inhibitor; ET, endocrine therapy; NR, not reported; Palbo, palbociclib; Ribo, ribociclib; RWE, real-world evidence.

#### Ribociclib

The RIBANNA study, reported in a conference abstract, compared the proportion of patients experiencing neutropenia between first-line ribociclib plus ET and ET alone, stratified by age subgroups (**Figure 3**, **Table 5**) (40). Among patients >80 years of age, the rates of overall and grade 3/4 neutropenia were lower compared to those aged 75-80, regardless of treatment. However, absolute neutropenia rates were notably higher with the ribociclib regimen (n=153-281) (overall: 20-23.7%; grade 3/4: 8.6-14.5%) compared to the ET control arm (n=41-42) (overall: 2.1-4.3%; grade 3/4: 0-4.3%) (40).

#### Abemaciclib

A single-arm study from the US, which adjusted for baseline characteristics, reported treatment discontinuation rates due to AEs across various age categories in patients receiving abemaciclib plus ET (**Figure 3**, **Table 5**) (41). The discontinuation rates increased with age, ranging from 19% in patients aged 18–49 years (n=42) to 48.4% in patients aged ≥85 years (n=31), with each successive age category showing progressively higher rates. This study was assessed as high-quality, with a NOS score of 8 (41).

#### Treatment patterns and safety outcomes of any CDK4/6i regimen in RWE studies

Three of the single-arm studies also reported treatment patterns and safety outcomes for any CDK4/6i regimens used in elderly and BIPOC subgroups (**Figure 3**, **Tables 6**, **7**) (34–36). Overall, higher discontinuation rates and neutropenia were reported for CDK4/6i in both subpopulations. Nevertheless, survival outcomes remained unaffected (**Tables 6**, **7**).

**Table 6:** Treatment patterns and safety outcomes for elderly patients receiving any CDK4/6i in RWE studies.

Study name; reference	Country	Treatment (Line of therapy)	Subgroup	Sample size	Treatment discontinuation, n (%)	Treatment discontinuation due to AEs, n (%)	Overall neutropenia, n (%)	Grade 3/4 neutropenia, n (%)
3585-Olazagasti-2023	US	CDK4/6i (All lines)	<70 years of age	129	NR	NR	49 (56.3)	NR
			≥70 years of age	73	NR	NR	20 (46.5)	NR
606-Fountzilas-2020	Greece	Palbo or ribo + ET (All lines)	>75 years old	43	NR	NR	17 (39.5)	8 (18.6)

AE, adverse event; CDK4/6i, cyclin-dependent kinase 4/6 inhibitor; ET, endocrine therapy; NR, not reported; Palbo, palbociclib; Ribo, ribociclib; RWE, real-world evidence; US, United States.

**Table 7:** Treatment patterns and safety outcomes for BIPOC patients receiving any CDK4/6i in RWE studies.

Study name; reference	Country	Treatment (Line of therapy)	Subgroup	Sample size	Treatment discontinuation, n (%)	Treatment discontinuation due to AEs, n (%)	Overall neutropenia, n (%)	Grade 3/4 neutropenia, n (%)
1198-Schreier-2022	US	CDK4/6i + Let, Ful, Ana, Exe or Tam (All lines)	Black patients	83	57 (68.67)	Neutropenia: 1 (1.20) Infection: 1 (1.20)	75 (90)	52 (63)
			Non-Black patients	99	57 (57.58)	Neutropenia: 1 (1.01) Infection: 1 (1.01)	81 (82)	42 (42)

AE, adverse event; Ana, anastrozole; CDK4/6i, cyclin-dependent kinase 4/6 inhibitor; Exe, exemestane; Ful, fulvestrant; Let, letrozole; RWE, real-world evidence; Tam, tamoxifen; US, United States.

In one US study evaluating CDK4/6i plus ET, Black patients (n=83) had a higher overall treatment discontinuation rate compared to non-Black patients (n=99) (68.7% vs. 57.6%), though discontinuations due to AEs were similar between groups. Black patients also experienced higher rates of neutropenia (overall: 90%; grade 3/4: 63%) compared to non-Black patients (overall: 82%; grade 3/4: 42%) (35).

A second US study evaluating CDK4/6i monotherapy, found lower neutropenia rates in older patients (≥70 years) compared to younger patients (46.5% [n=73] vs. 56.3% [n=129], respectively) (36). Lastly, a study from Greece assessing palbociclib or ribociclib plus ET in patients >75 years (n=43) reported overall and grade 3/4 neutropenia rates of 39.5% and 18.6%, respectively (34).

## Discussion

The previously published SLR summarized the real-world effectiveness and safety of CDK4/6i therapy in patients with HR+/HER2- a/mBC (17). However, as the real-world effectiveness and safety data has historically been scarce for elderly and BIPOC subgroups, despite their significant disease burden in HR+/HER2- a/mBC, the previous SLR was also limited in its synthesis of these underrepresented populations (7–9). Therefore, the current SLR serves to address this critical gap by providing a more up-to-date understanding of CDK4/6i treatment outcomes in these subpopulations less commonly studied in RCTs.

These results are based on additional data from 23 unique studies reporting data for elderly and/or BIPOC patients spanning almost five years since previously published findings comprising 6127 elderly patients, 1396 BIPOC patients, and spanning 16 countries, with the most studies (i.e., nine) providing data from the US. Notably, RWE studies evaluating palbociclib (n=14) (19–29, 37–39) in HR+/HER2- a/mBC in the elderly and/or BIPOC populations were by far the most numerous compared with ribociclib (n=2) (30, 40) or abemaciclib (n=1) (41). Furthermore, only one study directly compared all three CDK4/6is head-to-head (31) and the remaining studies (n=5) evaluated CDK4/6i regimens collectively, without specifying results for individual inhibitors (32–36). Additionally, far fewer studies investigated subgroup analyses in BIPOC populations (n=3) (29, 33, 35) than in elderly patients (n=18) (21–28, 30–32, 34, 36–41), with two studies evaluating both subgroups (19, 20). All five available BIPOC studies evaluated either palbociclib or a generalized CDK4/6i regimen. This relative disparity in subgroup-specific RWE, particularly for BIPOC patients, available for ribociclib and abemaciclib can most likely be attributed to these agents being approved more recently in the US than palbociclib. Further research into this underrepresented group remains an opportunity as more evidence emerges from ongoing and upcoming RWE CDK4/6i studies.

Seventeen studies were assessed for quality using the NOS assessment tool. Among these, six were classified as high-quality (26, 28, 29, 31, 33, 41), while 11 were of moderate-quality (19–25, 34–37). All studies were either truly or partially representative of the exposed patient cohort, confirmed that the outcomes of interest were not present at the study’s onset, and obtained patient data from surgical records. While most studies did not compare treatments between cohorts, those that did employed robust methods such as multivariate analysis, stabilized inverse probability of treatment weights, or propensity-score matching (26, 28, 29, 33). Independent blind assessment was used to evaluate efficacy outcomes in all studies. Median follow-up times were reported in 13 studies, ranging from 7.4 to 49.5 months (20, 35); however, four of these lacked sufficient follow-up duration for efficacy outcomes to be observed (20, 22, 34, 41). Notably, many studies included in the review did not provide patient follow-up statements, with only one study reporting no loss to follow-up (21) and three reporting an adequate follow-up rate (>10%) (25, 34, 35).

### Elderly populations

Although comparisons between elderly and younger patients with HR+/HER2- a/mBC were not consistent across all eligible studies, the collective evidence suggests that CDK4/6i are generally effective in patients aged ≥65 years. In both single-arm studies and comparative studies of palbociclib versus ET, palbociclib-based regimens demonstrated consistent improvements in PFS and OS for elderly patients, particularly in the first-line setting with AI combinations (21–23, 26, 28, 29). However, some variations were observed, with shorter PFS or OS reported in older subgroups, particularly those aged ≥70 or ≥80 years (23, 31) When all lines were considered, PFS and OS rates were comparable between younger and older patients (20). These data are aligned with a recent SLR by Brain and colleagues, which highlighted the efficacy and tolerability of palbociclib in elderly patients, with comparable clinical benefits and quality of life to younger populations (5). However, the review focused solely on palbociclib. Ribociclib plus AI or fulvestrant demonstrated similar median PFS between patients ≥75 years and younger patients in the first-line setting in one single-arm study (30).

In the only study to directly compare all three CDK4/6 inhibitors, Tang et al. observed notable differences in outcomes by age group (31). Palbociclib demonstrated the most favorable outcomes in patients aged ≤65 years and 66–70 years, with the longest PFS and OS compared to ribociclib and abemaciclib. For patients aged ≥80 years, ribociclib achieved the best results; however, the generalizability of these findings may be limited due to the small sample size of patients in the ribociclib cohort (n=5). Further, ribociclib was approved for use in the US more recently than palbociclib, resulting in fewer long-term real-world studies assessing survival outcomes in patients with HR+/HER2- a/mBC, particularly in elderly and BIPOC patients. Although it should be noted that these studies are limited by their descriptive design. Emerging RWE for ribociclib from ongoing and upcoming studies with larger cohorts will better inform future comparisons of effectiveness outcomes with palbociclib in these subgroups.

Five studies evaluated treatment patterns and/or safety outcomes in elderly patients with HR+/HER2- a/mBC (37–41). Palbociclib showed similar rates of treatment discontinuation across age subgroups (37–39), whereas discontinuation rates of abemaciclib increased steadily with age, with the highest rates seen in patients aged ≥85 years (41). Limited data for ribociclib indicated higher rates of neutropenia in patients aged 75–80 years compared to those aged >80 years and higher overall rates with ribociclib regimens than with ET alone (40). However, the results from these studies are limited due to the small sample sizes and solely descriptive study design. The studies included in this review also did not highlight any new AEs, supporting the overall reported safety of using CDK4/6i treatments in elderly patients with HR+/HER2- a/mBC.

### BIPOC populations

Evidence regarding racial and ethnic differences in CDK4/6i outcomes remains sparse, with no available data on treatment patterns or safety outcomes for BIPOC patient subgroups. Of the five studies reporting effectiveness outcomes, one of these studies demonstrated improvements in both PFS and OS when treated with palbociclib plus AI versus with AI alone (28). Similarly, following treatment with palbocilib+AI, the IRIS study reported the highest rates of PFS and OS among the Middle Eastern patient group (90.9%) and the ‘Other’ patient group, respectively (20). However, within the palbociclib+fulvestrant treatment group, PFS and OS rates were lowest among White patients (45.3% and 86.5%, respectively). This contrasts with two studies demonstrating consistent improvements in median PFS among White patients compared to Black or African American patients (19, 35). This finding aligns with an analysis by Knudsen et al., which reported that African American patients received treatment with CDK4/6i more frequently with recurrent disease and in combination with fulvestrant, both of which are associated with shorter PFS (43). However, of the study’s non-European patient cohort, those of African descent only accounted for approximately 8% of patients, further emphasizing the current limited understanding of how race and ethnicity influence CDK4/6i treatment responses.

### Future research

Although the current body evidence for CDK4/6i effectiveness data in elderly and BIPOC patient subgroups with HR+/HER2- a/mBC is evolving, there are significant geographic disparities to be addressed. Future studies may consider evaluating, treatment patterns, and/or safety outcomes in more diverse patient populations across Asia, Latin America, and the Middle East to address existing racial/ethnic disparities in HR+/HER2- a/mBC treatment. Another area of exploration may involve determining how different comorbidities influence responses to CDK4/6i within high-risk patient subgroups, like the elderly, as well as capturing any geographic variations in these trends.

Moreover, there was a lack of standardization with regards to the threshold definition for elderly patients, which ranged anywhere from ≥65 to ≥80 years of age. There is an opportunity for a wider analysis of different elderly age groups to determine at which range patients can best derive benefits from each CDK4/6i. To fully understand the impact of treatment for advanced/metastatic breast cancer, it is important to not only assess efficacy and safety but also patients’ quality of life and experiences while on treatment. We have previously reported on PRO and HRQoL data in patients with HR+/HER2- aBC or mBC treated with palbociclib (44). Here we synthesized RCT and RWE data, and the evidence largely supported the preservation of quality of life with the addition of the CDK4/6i, palbociclib, to endocrine therapy in patients with HR+/HER2- aBC or mBC. A separate SLR synthesizing clinical trial and real-world evidence for palbociclib treatment outcomes in older patients (≥ 65 years for RCTs; > 60 years for RWE) with HR+/HER2- a/mBC, also showed that global QoL was maintained in older patients receiving palbociclib (5). A challenge remains with RWE that quality of life data is rarely reported. Furthermore, the main source of heterogeneity across RCT and those RWE studies that report quality life data is the use of different HRQoL instruments and lack of consistent application across studies presenting a barrier in comparing results between studies. Nonetheless, RWE can offer valuable data, especially on PROs in a real-world population. Future studies may consider exploring PRO and HRQoL that are not addressed in the scope of the current review in the elderly and BIPOC or other patient populations. Continued focused efforts should be made to incorporate PRO and HRQoL assessments more widely in treatment evaluations and clinical practice moving forward. Another potential direction for future research to consider is incorporating more patient-reported outcomes in RWE studies to better understand the real-world impact of CDK4/6i on quality of life, particularly in underrepresented subgroups and across different lines of therapy. There is also a need for additional comparative effectiveness studies between the CDK4/6is to better differentiate between treatment options for eligible patients, as well as greater RWE of the role of socioeconomic factors in CDK4/6i outcomes in elderly and BIPOC populations.

### Study limitations

This review has some limitations. Given a limited body of RWE evaluating CDK4/6i outcomes in the elderly and especially in BIPOC populations, the decision to exclude studies with fewer than 100 patients in the current synthesis may have removed additional data potentially relevant to the outcomes and subgroups of interest. Accordingly, this review captured no data illustrating CDK4/6i effectiveness for ribociclib and abemaciclib in BIPOC patients with HR+/HER2- a/mBC. Further, Black or African American patients were the only subgroup with data available for efficacy, treatment patterns, and safety outcomes. In contrast, other subgroups, such as Asian, Hispanic, and Native American populations, only had efficacy data. This further highlights a gap in the knowledge base of understanding CDK4/6i treatment patterns and safety outcomes in these underrepresented racial/ethnic groups. Owing to palbociclib being approved earlier than these two CDK4/6i, the majority of included studies also only evaluated palbociclib regimens in older patients, with no effectiveness data available for the clinical use of abemaciclib in this subgroup. However, this is likely to change as more long-term follow-up data emerges from ongoing studies for ribociclib and abemaciclib for patients in these subgroups. Across the CDK4/6i class, there were also limited data assessing safety and treatment patterns in the subpopulations of interest. These gaps indicate additional studies are required to further validate the interpretations and findings reported in the current review. Lastly, we acknowledge that the latest update of the systematic literature review was performed in January 2024. While we strive to include the most recent and relevant studies, we recognize that the dynamic nature of research means that literature reviews can never be entirely up-to-date. However, we have made every effort to incorporate the latest data available at the time of writing. We also acknowledge that this is an area that will benefit from continuous updates as new research emerges.

Although the current understanding of CDK4/6i outcomes in elderly and BIPOC patients is still in its early stages, with additional data for newer agents continuing to emerge, these results offer valuable insights for shaping the future of CDK4/6i therapy in HR+/HER2- a/mBC. Certain subgroups like older adults and BIPOC patients are known to be underrepresented in breast cancer clinical trials despite carrying significant disease burden (6–9). Conducting targeted analysis of available RWE in these populations has the potential to better guide clinical decision making by revealing characteristic differences between individuals who experience sustained long-term benefits from CDK4/6i therapy and those with short-lived treatment responses. The current review offers insights which may help refine patient selection in real-world clinical settings, improve AE management in underrepresented patient groups, and better enable clinicians to proactively tailor their treatment strategies for HR+/HER2- a/mBC.

## Conclusion

This review demonstrated that CDK4/6is are effective and are generally well-tolerated in elderly and BIPOC patients with HR+/HER2- a/mBC. The observed clinical benefits in these populations in real-world settings are consistent with findings from prior reviews and clinical trials, reinforcing the broader applicability of CDK4/6is across diverse patient groups and significant role in informing real-world clinical decision-making.

## Data Availability

The original contributions presented in the study are included in the article/**Supplementary Material**. Further inquiries can be directed to the corresponding author.
