# Supplementary material for: Real-world effectiveness and safety of CDK4/6i in elderly and BIPOC patients with HR+/HER2- advanced/metastatic breast cancer: an updated systematic literature review
Source: Front Oncol. 2025 Aug 15;15:1577075. doi: 10.3389/fonc.2025.1577075 (PMC12394504; doi:10.3389/fonc.2025.1577075)
Supplement: Supplementary file 1 [file SupplementaryFile1.docx]

Supplementary Material

# PRISMA 2020 Checklist

| **Section and Topic** | **Item #** | **Checklist item** | **Location where item is reported** |
| --- | --- | --- | --- |
| **TITLE** | | |  |
| Title | 1 | Identify the report as a systematic review. | Title Page |
| **ABSTRACT** | | |  |
| Abstract | 2 | See the PRISMA 2020 for Abstracts checklist. | Abstract |
| **INTRODUCTION** | | |  |
| Rationale | 3 | Describe the rationale for the review in the context of existing knowledge. | Introduction |
| Objectives | 4 | Provide an explicit statement of the objective(s) or question(s) the review addresses. | Introduction |
| **METHODS** | | |  |
| Eligibility criteria | 5 | Specify the inclusion and exclusion criteria for the review and how studies were grouped for the syntheses. | Materials and Methods: Study Selection and Data Extraction |
| Information sources | 6 | Specify all databases, registers, websites, organisations, reference lists and other sources searched or consulted to identify studies. Specify the date when each source was last searched or consulted. | Materials and Methods: Literature Search |
| Search strategy | 7 | Present the full search strategies for all databases, registers and websites, including any filters and limits used. | Supplementary Material |
| Selection process | 8 | Specify the methods used to decide whether a study met the inclusion criteria of the review, including how many reviewers screened each record and each report retrieved, whether they worked independently, and if applicable, details of automation tools used in the process. | Materials and Methods: Study Selection and Data Extraction |
| Data collection process | 9 | Specify the methods used to collect data from reports, including how many reviewers collected data from each report, whether they worked independently, any processes for obtaining or confirming data from study investigators, and if applicable, details of automation tools used in the process. | Materials and Methods: Study Selection and Data Extraction |
| Data items | 10a | List and define all outcomes for which data were sought. Specify whether all results that were compatible with each outcome domain in each study were sought (e.g. for all measures, time points, analyses), and if not, the methods used to decide which results to collect. | Materials and Methods: Data Analysis |
|  | 10b | List and define all other variables for which data were sought (e.g. participant and intervention characteristics, funding sources). Describe any assumptions made about any missing or unclear information. | Materials and Methods: Data Analysis |
| Study risk of bias assessment | 11 | Specify the methods used to assess risk of bias in the included studies, including details of the tool(s) used, how many reviewers assessed each study and whether they worked independently, and if applicable, details of automation tools used in the process. | Materials and Methods: Quality Assessment |
| Effect measures | 12 | Specify for each outcome the effect measure(s) (e.g. risk ratio, mean difference) used in the synthesis or presentation of results. | - |
| Synthesis methods | 13a | Describe the processes used to decide which studies were eligible for each synthesis (e.g. tabulating the study intervention characteristics and comparing against the planned groups for each synthesis (item #5)). | Methods: Study Selection and Data Extraction |
|  | 13b | Describe any methods required to prepare the data for presentation or synthesis, such as handling of missing summary statistics, or data conversions. | Methods: Study Selection and Data Extraction |
|  | 13c | Describe any methods used to tabulate or visually display results of individual studies and syntheses. | Methods: Study Selection and Data Extraction |
|  | 13d | Describe any methods used to synthesize results and provide a rationale for the choice(s). If meta-analysis was performed, describe the model(s), method(s) to identify the presence and extent of statistical heterogeneity, and software package(s) used. | Methods: Study Selection and Data Extraction |
|  | 13e | Describe any methods used to explore possible causes of heterogeneity among study results (e.g. subgroup analysis, meta-regression). | - |
|  | 13f | Describe any sensitivity analyses conducted to assess robustness of the synthesized results. | - |
| Reporting bias assessment | 14 | Describe any methods used to assess risk of bias due to missing results in a synthesis (arising from reporting biases). | - |
| Certainty assessment | 15 | Describe any methods used to assess certainty (or confidence) in the body of evidence for an outcome. | - |
| **RESULTS** | | |  |
| Study selection | 16a | Describe the results of the search and selection process, from the number of records identified in the search to the number of studies included in the review, ideally using a flow diagram. | Results: Literature search & Study Selection, Figure 1, Supplementary Material |
|  | 16b | Cite studies that might appear to meet the inclusion criteria, but which were excluded, and explain why they were excluded. | - |
| Study characteristics | 17 | Cite each included study and present its characteristics. | Results: Literature search & Study Selection, Effectiveness of CDK4/6i in RWE Studies |
| Risk of bias in studies | 18 | Present assessments of risk of bias for each included study. | Results: Quality Assessment, Effectiveness of CDK4/6i in RWE Studies, Supplementary Material |
| Results of individual studies | 19 | For all outcomes, present, for each study: (a) summary statistics for each group (where appropriate) and (b) an effect estimate and its precision (e.g. confidence/credible interval), ideally using structured tables or plots. | Results: Effectiveness of CDK4/6i in RWE Studies, Tables 1-7 |
| Results of syntheses | 20a | For each synthesis, briefly summarise the characteristics and risk of bias among contributing studies. | Results: Effectiveness of CDK4/6i in RWE Studies |
|  | 20b | Present results of all statistical syntheses conducted. If meta-analysis was done, present for each the summary estimate and its precision (e.g. confidence/credible interval) and measures of statistical heterogeneity. If comparing groups, describe the direction of the effect. | - |
|  | 20c | Present results of all investigations of possible causes of heterogeneity among study results. | Results: Effectiveness of CDK4/6i in RWE Studies |
|  | 20d | Present results of all sensitivity analyses conducted to assess the robustness of the synthesized results. | - |
| Reporting biases | 21 | Present assessments of risk of bias due to missing results (arising from reporting biases) for each synthesis assessed. | - |
| Certainty of evidence | 22 | Present assessments of certainty (or confidence) in the body of evidence for each outcome assessed. | - |
| **DISCUSSION** | | |  |
| Discussion | 23a | Provide a general interpretation of the results in the context of other evidence. | Discussion |
|  | 23b | Discuss any limitations of the evidence included in the review. | Discussion: Study Limitations |
|  | 23c | Discuss any limitations of the review processes used. | Discussion: Study Limitations |
|  | 23d | Discuss implications of the results for practice, policy, and future research. | Discussion: Future Research |
| **OTHER INFORMATION** | | |  |
| Registration and protocol | 24a | Provide registration information for the review, including register name and registration number, or state that the review was not registered. | - |
|  | 24b | Indicate where the review protocol can be accessed, or state that a protocol was not prepared. | - |
|  | 24c | Describe and explain any amendments to information provided at registration or in the protocol. | - |
| Support | 25 | Describe sources of financial or non-financial support for the review, and the role of the funders or sponsors in the review. | Funding |
| Competing interests | 26 | Declare any competing interests of review authors. | Conflict of Interest |
| Availability of data, code and other materials | 27 | Report which of the following are publicly available and where they can be found: template data collection forms; data extracted from included studies; data used for all analyses; analytic code; any other materials used in the review. | Data Availability Statement |

Abbreviations: N/A, not available.

*From:* Page MJ, McKenzie JE, Bossuyt PM, Boutron I, Hoffmann TC, Mulrow CD, et al. The PRISMA 2020 statement: an updated guideline for reporting systematic reviews. BMJ 2021;372:n71. doi: 10.1136/bmj.n71

For more information, visit: <http://www.prisma-statement.org/>

# Most Recent Search Strategy

## January 9, 2024 Update

**Date of the search:** 9 Jan 2024

**Update date span:** Oct 2023 - Current (based on the update dates of the included databases, CENTRAL & CDSR update: Sept 2022 - Current)

**Databases searched:**

Ovid MEDLINE(R) and Epub Ahead of Print, In-Process & Other Non-Indexed Citations and Daily

Ovid EMBASE

Ovid EBM Reviews - Cochrane Central Register of Controlled Trials

Ovid EBM Reviews - Cochrane Database of Systematic Reviews

**Summary of Results:**

| **Search** | **Ovid Results** | **Results deduped in Ovid** | **Results deduped in EndNote** |
| --- | --- | --- | --- |
| CDK4/6i SLR Update Oct 2023 - Current | 708 | 578 | 572 |

Search history saved in **Corner3** as: "Breast Cancer - Palbociclib - Multifile - UPDATE 6 -120 000443- FINAL v3"

09 Jan 2024 - UPDATE #6 timespan: Oct 2023 (Sept 2022 for CENTRAL & CDSR) - Current

**MULTIFILE SEARCH**

Database(s): **EBM Reviews - Cochrane Central Register of Controlled Trials**December 2023**, EBM Reviews - Cochrane Database of Systematic Reviews**2005 to January 3, 2024**, Embase**1974 to 2024 January 08**, Ovid MEDLINE(R) and Epub Ahead of Print, In-Process, In-Data-Review & Other Non-Indexed Citations and Daily**1946 to January 08, 2024

Search Strategy:

| **#** | **Searches** | **Results** |
| --- | --- | --- |
| 1 | exp Breast Neoplasms/ | 1029163 |
| 2 | exp Breast Carcinoma In Situ/ | 15846 |
| 3 | ((breast$1 or mamma or mammary) adj3 (adenocarcinoma* or cancer* or carcinoma* or neoplasm* or tumour* or tumor*)).tw,kf. | 1085445 |
| 4 | ((ductal or intraductal or intra-ductal) adj (carcinoma? or hyperplasia?)).tw,kf. | 49610 |
| 5 | DCIS.tw,kf. | 18207 |
| 6 | (lobul* carcinoma? adj2 "in situ").tw,kf. | 2992 |
| 7 | LCIS.tw,kf. | 1951 |
| 8 | (paget* and (areola? or breast* or mammary or nipple*)).tw,kf. | 3442 |
| 9 | or/1-8 [BREAST CANCER] | 1309948 |
| 10 | Receptor, ErbB-2/ | 107636 |
| 11 | ERBB2 protein, human.nm. | 12851 |
| 12 | (ErbB2 or "ErbB 2" or HER2* or "HER 2*" or "c-ErbB2" or "C-ErbB 2").tw,kf. | 165534 |
| 13 | ((oncoprotein* or onco-protein* or protein* or receptor*) adj1 (neu or neuregulin)).tw,kf. | 3235 |
| 14 | CD340.tw,kf. | 49 |
| 15 | ("p185(c-neu)" or p185erbB).tw,kf. | 90 |
| 16 | (neu protooncogene or neu proto-oncogene).tw,kf. | 454 |
| 17 | NGL.tw,kf. | 1962 |
| 18 | metastatic lymph node gene 19.tw,kf. | 0 |
| 19 | (MLN19 or MLN 19).tw,kf. | 2 |
| 20 | (human adj1 "epidermal growth factor receptor 2").tw,kf. | 27941 |
| 21 | ErbB Receptors/ | 156368 |
| 22 | or/10-21 | 333450 |
| 23 | 9 and 22 [HER2 BREAST CANCER] | 149931 |
| 24 | exp Receptors, Estrogen/ | 169215 |
| 25 | estrogen receptor?.tw,kf. | 146412 |
| 26 | oestrogen receptor?.tw,kf. | 16112 |
| 27 | ERalpha*.tw,kf. | 29345 |
| 28 | ERbeta.tw,kf. | 15152 |
| 29 | ER positive.tw,kf. | 20841 |
| 30 | "luminal a".tw,kf. | 10276 |
| 31 | "luminal b".tw,kf. | 8689 |
| 32 | (luminal adj2 subtype*).tw,kf. | 6495 |
| 33 | hormone receptor?.tw,kf. | 93480 |
| 34 | HR positive.tw,kf. | 4531 |
| 35 | progesterone receptor?.tw,kf. | 59555 |
| 36 | progestin receptor?.tw,kf. | 1925 |
| 37 | PR positive.tw,kf. | 4286 |
| 38 | or/24-37 | 332470 |
| 39 | 9 and 38 [HR BREAST CANCER] | 169470 |
| 40 | exp Breast Neoplasms/sc [secondary/metastatic] | 5262 |
| 41 | (advanced adj3 ((breast$1 or mamma or mammary) adj3 (adenocarcinoma* or cancer* or carcinoma* or neoplasm* or tumour* or tumor*))).tw,kf. | 40140 |
| 42 | (metastatic* adj3 ((breast$1 or mamma or mammary) adj3 (adenocarcinoma* or cancer* or carcinoma* or neoplasm* or tumour* or tumor*))).tw,kf. | 73332 |
| 43 | (metastas* adj3 ((breast$1 or mamma or mammary) adj3 (adenocarcinoma* or cancer* or carcinoma* or neoplasm* or tumour* or tumor*))).tw,kf. | 39821 |
| 44 | (progressive adj3 ((breast$1 or mamma or mammary) adj3 (adenocarcinoma* or cancer* or carcinoma* or neoplasm* or tumour* or tumor*))).tw,kf. | 658 |
| 45 | (secondary adj3 ((breast$1 or mamma or mammary) adj3 (adenocarcinoma* or cancer* or carcinoma* or neoplasm* or tumour* or tumor*))).tw,kf. | 2803 |
| 46 | (terminal adj3 ((breast$1 or mamma or mammary) adj3 (adenocarcinoma* or cancer* or carcinoma* or neoplasm* or tumour* or tumor*))).tw,kf. | 171 |
| 47 | or/40-46 [METASTATIC BREAST CANCER] | 140337 |
| 48 | 23 or 39 or 47 [BREAST CANCERS OF INTEREST] | 344074 |
| 49 | exp Infant/ not exp Adult/ | 1857551 |
| 50 | exp Child/ not exp Adult/ | 3735929 |
| 51 | Adolescent/ not exp Adult/ | 1408289 |
| 52 | 48 not (49 or 50 or 51) [INFANT-, CHILD-, ADOLESCENT-ONLY REMOVED] | 343239 |
| 53 | Palbociclib.nm. | 956 |
| 54 | (palbociclib or ibrance$2 or palbociclib or "pd 0332991" or "pd 0332991" or pd332991or pd332991 or "pf 00080665" or pf00080665).tw,kf. | 7202 |
| 55 | ribociclib.nm. | 344 |
| 56 | (ribociclib or kisqali$2 or "lee 011" or lee 011a or lee 011bba or lee 11 or lee 11a or lee 11bba or lee011 or lee011a or lee011bba or lee11 or lee11a or lee11bba).tw,kf. | 3050 |
| 57 | (abemaciclib or bemaciclib$2 or ly 2835219 or ly2835219 or verzenio$2).tw,kf. | 3046 |
| 58 | Cyclin-Dependent Kinase Inhibitor Proteins/ | 9206 |
| 59 | cyclin-dependent kinase inhibitor?.tw,kf. | 16182 |
| 60 | (CDKI or CDKIs).tw,kf. | 1532 |
| 61 | CKI Protein?.tw,kf. | 47 |
| 62 | (CIP-KIP adj2 protein?).tw,kf. | 85 |
| 63 | Cyclin-Dependent Kinases/ | 22674 |
| 64 | Cyclin-Dependent Kinase 4/ | 20275 |
| 65 | Cyclin-Dependent Kinase 6/ | 11962 |
| 66 | (Cyclin-Dependent Kinase 4 or Cyclin-Dependent Kinase 6).tw,kf. | 6075 |
| 67 | (CDK4* or CDK 4* or CDK6* or CDK 6*).tw,kf. | 32198 |
| 68 | (Cell Division Protein Kinase 4 or PSK-J3 Kinase or PSKJ3 Kinase or p34PSK-J3 Kinase or p34PSKJ3 Kinase).tw,kf. | 6 |
| 69 | (Cell Division Protein Kinase 6 or PLSTIRE Protein).tw,kf. | 12 |
| 70 | or/53-69 [FIRST LINE DRUGS] | 78591 |
| 71 | 52 and 70 [BREAST CANCERS OF INTEREST - FIRST LINE DRUGS] | 10739 |
| 72 | exp Animals/ not Humans/ | 16902871 |
| 73 | 71 not 72 [ANIMAL-ONLY REMOVED] | 9919 |
| 74 | limit 73 to yr="2023-current" | 1928 |
| 75 | 74 use ppez [MEDLINE RECORDS] | 523 |
| 76 | exp breast cancer/ | 948461 |
| 77 | ((breast$1 or mamma or mammary) adj3 (adenocarcinoma* or cancer* or carcinoma* or neoplasm* or tumour* or tumor*)).tw,kw. | 1070247 |
| 78 | ((ductal or intraductal or intra-ductal) adj (carcinoma? or hyperplasia?)).tw,kw. | 48962 |
| 79 | DCIS.tw,kw. | 18130 |
| 80 | (lobul* carcinoma? adj2 "in situ").tw,kw. | 2920 |
| 81 | LCIS.tw,kw. | 1942 |
| 82 | (paget* and (areola? or breast* or mammary or nipple*)).tw,kw. | 3455 |
| 83 | or/76-82 [BREAST CANCER] | 1279169 |
| 84 | epidermal growth factor receptor 2/ | 80055 |
| 85 | (ErbB2 or "ErbB 2" or HER2* or "HER 2*" or "c-ErbB2" or "C-ErbB 2").tw,kw. | 165364 |
| 86 | ((oncoprotein* or onco-protein* or protein* or receptor*) adj1 (neu or neuregulin)).tw,kw. | 3223 |
| 87 | CD340.tw,kw. | 49 |
| 88 | ("p185(c-neu)" or p185erbB).tw,kw. | 90 |
| 89 | (neu protooncogene or neu proto-oncogene).tw,kw. | 453 |
| 90 | NGL.tw,kw. | 1957 |
| 91 | metastatic lymph node gene 19 protein*.tw,kw. | 0 |
| 92 | (MLN19 or MLN 19).tw,kw. | 2 |
| 93 | (human adj1 "epidermal growth factor receptor 2").tw,kw. | 28093 |
| 94 | or/84-93 | 193980 |
| 95 | 83 and 94 [HER2 BREAST CANCER] | 137033 |
| 96 | exp estrogen receptor/ | 169215 |
| 97 | estrogen receptor?.tw,kw. | 145488 |
| 98 | oestrogen receptor?.tw,kw. | 15985 |
| 99 | ERalpha*.tw,kw. | 29320 |
| 100 | ERbeta.tw,kw. | 15122 |
| 101 | exp estrogen receptor positive breast cancer/ | 12198 |
| 102 | ER positive.tw,kw. | 20788 |
| 103 | "luminal a".tw,kw. | 10275 |
| 104 | "luminal b".tw,kw. | 8685 |
| 105 | (luminal adj2 subtype*).tw,kw. | 6441 |
| 106 | hormone receptor?.tw,kw. | 91091 |
| 107 | progesterone receptor positive breast cancer/ | 2165 |
| 108 | HR positive.tw,kw. | 4497 |
| 109 | progesterone receptor?.tw,kw. | 59314 |
| 110 | PR positive.tw,kw. | 4276 |
| 111 | progestin receptor?.tw,kw. | 1919 |
| 112 | or/96-111 | 332793 |
| 113 | 83 and 112 [HR BREAST CANCER] | 171392 |
| 114 | metastatic breast cancer/ | 17223 |
| 115 | (advanced adj3 ((breast$1 or mamma or mammary) adj3 (adenocarcinoma* or cancer* or carcinoma* or neoplasm* or tumour* or tumor*))).tw,kw. | 39888 |
| 116 | (metastatic* adj3 ((breast$1 or mamma or mammary) adj3 (adenocarcinoma* or cancer* or carcinoma* or neoplasm* or tumour* or tumor*))).tw,kw. | 72798 |
| 117 | (metastas* adj3 ((breast$1 or mamma or mammary) adj3 (adenocarcinoma* or cancer* or carcinoma* or neoplasm* or tumour* or tumor*))).tw,kw. | 40009 |
| 118 | (progressive adj3 ((breast$1 or mamma or mammary) adj3 (adenocarcinoma* or cancer* or carcinoma* or neoplasm* or tumour* or tumor*))).tw,kw. | 658 |
| 119 | (secondary adj3 ((breast$1 or mamma or mammary) adj3 (adenocarcinoma* or cancer* or carcinoma* or neoplasm* or tumour* or tumor*))).tw,kw. | 2770 |
| 120 | (terminal adj3 ((breast$1 or mamma or mammary) adj3 (adenocarcinoma* or cancer* or carcinoma* or neoplasm* or tumour* or tumor*))).tw,kw. | 174 |
| 121 | or/114-120 [METASTATIC BREAST CANCER] | 140684 |
| 122 | 95 or 113 or 121 [BREAST CANCERS OF INTEREST] | 336637 |
| 123 | adolescent/ not exp adult/ | 1408289 |
| 124 | child/ not exp adult/ | 2699095 |
| 125 | infant/ not exp adult/ | 1176104 |
| 126 | fetus/ not exp adult/ | 244311 |
| 127 | 122 not (123 or 124 or 125 or 126) [FETUS-, INFANT-, CHILD-, ADOLESCENT-ONLY REMOVED] | 335859 |
| 128 | palbociclib/ | 7133 |
| 129 | (palbociclib or ibrance$2 or palbociclib or "pd 0332991" or "pd 0332991" or pd332991or pd332991 or "pf 00080665" or pf00080665).tw,kw. | 7196 |
| 130 | ribociclib/ | 2921 |
| 131 | (ribociclib or kisqali$2 or "lee 011" or lee 011a or lee 011bba or lee 11 or lee 11a or lee 11bba or lee011 or lee011a or lee011bba or lee11 or lee11a or lee11bba).tw,kw. | 3045 |
| 132 | abemaciclib/ | 3120 |
| 133 | (abemaciclib or bemaciclib$2 or ly 2835219 or ly2835219 or verzenio$2).tw,kw. | 3042 |
| 134 | cyclin dependent kinase inhibitor/ | 8439 |
| 135 | cyclin-dependent kinase inhibitor?.tw,kw. | 15939 |
| 136 | (CDKI or CDKIs).tw,kw. | 1512 |
| 137 | CKI protein?.tw,kw. | 47 |
| 138 | (CIP-KIP adj2 protein?).tw,kw. | 84 |
| 139 | cyclin dependent kinase/ | 23120 |
| 140 | cyclin dependent kinase 4/ | 20275 |
| 141 | cyclin dependent kinase 6/ | 11962 |
| 142 | (Cyclin-Dependent Kinase 4 or Cyclin-Dependent Kinase 6).tw,kw. | 5974 |
| 143 | (CDK4* or CDK 4* or CDK6* or CDK 6*).tw,kw. | 32182 |
| 144 | (Cell Division Protein Kinase 4 or PSK-J3 Kinase or PSKJ3 Kinase or p34PSK-J3 Kinase or p34PSKJ3 Kinase).tw,kw. | 6 |
| 145 | (Cell Division Protein Kinase 6 or PLSTIRE Protein).tw,kw. | 12 |
| 146 | or/128-145 [FIRST LINE DRUGS] | 80700 |
| 147 | 127 and 146 [BREAST CANCERS OF INTEREST - FIRST LINE DRUGS] | 11469 |
| 148 | exp animal/ or exp animal experimentation/ or exp animal model/ or exp animal experiment/ or nonhuman/ or exp vertebrate/ | 61019819 |
| 149 | exp human/ or exp human experimentation/ or exp human experiment/ | 48486809 |
| 150 | 148 not 149 | 12535021 |
| 151 | 147 not 150 [ANIMAL-ONLY REMOVED] | 11208 |
| 152 | limit 151 to yr="2023-current" | 2090 |
| 153 | 152 use oemezd [EMBASE RECORDS] | 1412 |
| 154 | exp Breast Neoplasms/ | 1029163 |
| 155 | exp Breast Carcinoma In Situ/ | 15846 |
| 156 | ((breast$1 or mamma or mammary) adj3 (adenocarcinoma* or cancer* or carcinoma* or neoplasm* or tumour* or tumor*)).ti,ab,kw. | 1069857 |
| 157 | ((ductal or intraductal or intra-ductal) adj (carcinoma? or hyperplasia?)).ti,ab,kw. | 48936 |
| 158 | DCIS.ti,ab,kw. | 18110 |
| 159 | (lobul* carcinoma? adj2 "in situ").ti,ab,kw. | 2909 |
| 160 | LCIS.ti,ab,kw. | 1934 |
| 161 | (paget* and (areola? or breast* or mammary or nipple*)).ti,ab,kw. | 3445 |
| 162 | or/154-161 [BREAST CANCER] | 1304645 |
| 163 | Receptor, ErbB-2/ | 107636 |
| 164 | (ERBB2 protein adj1 human).mp. | 14454 |
| 165 | (ErbB2 or "ErbB 2" or HER2* or "HER 2*" or "c-ErbB2" or "C-ErbB 2").ti,ab,kw. | 165301 |
| 166 | ((oncoprotein* or onco-protein* or protein* or receptor*) adj1 (neu or neuregulin)).ti,ab,kw. | 3220 |
| 167 | CD340.ti,ab,kw. | 49 |
| 168 | ("p185(c-neu)" or p185erbB).ti,ab,kw. | 90 |
| 169 | (neu protooncogene or neu proto-oncogene).ti,ab,kw. | 453 |
| 170 | NGL.ti,ab,kw. | 1955 |
| 171 | metastatic lymph node gene 19.ti,ab,kw. | 0 |
| 172 | (MLN19 or MLN 19).ti,ab,kw. | 2 |
| 173 | (human adj1 "epidermal growth factor receptor 2").ti,ab,kw. | 28069 |
| 174 | ErbB Receptors/ | 156368 |
| 175 | or/163-174 | 333294 |
| 176 | 162 and 175 [HER2 BREAST CANCER] | 149628 |
| 177 | exp Receptors, Estrogen/ | 169215 |
| 178 | estrogen receptor?.ti,ab,kw. | 145456 |
| 179 | oestrogen receptor?.ti,ab,kw. | 15912 |
| 180 | ERalpha*.ti,ab,kw. | 29320 |
| 181 | ERbeta.ti,ab,kw. | 15120 |
| 182 | ER positive.ti,ab,kw. | 20770 |
| 183 | "luminal a".ti,ab,kw. | 10271 |
| 184 | "luminal b".ti,ab,kw. | 8681 |
| 185 | (luminal adj2 subtype*).ti,ab,kw. | 6439 |
| 186 | hormone receptor?.ti,ab,kw. | 91040 |
| 187 | HR positive.ti,ab,kw. | 4495 |
| 188 | progesterone receptor?.ti,ab,kw. | 59252 |
| 189 | progestin receptor?.ti,ab,kw. | 1918 |
| 190 | PR positive.ti,ab,kw. | 4271 |
| 191 | or/177-190 | 329702 |
| 192 | 162 and 191 [HR BREAST CANCER] | 168871 |
| 193 | exp Breast Neoplasms/sc [secondary/metastatic] | 5262 |
| 194 | (advanced adj3 ((breast$1 or mamma or mammary) adj3 (adenocarcinoma* or cancer* or carcinoma* or neoplasm* or tumour* or tumor*))).ti,ab,kw. | 39846 |
| 195 | (metastatic* adj3 ((breast$1 or mamma or mammary) adj3 (adenocarcinoma* or cancer* or carcinoma* or neoplasm* or tumour* or tumor*))).ti,ab,kw. | 72757 |
| 196 | (metastas* adj3 ((breast$1 or mamma or mammary) adj3 (adenocarcinoma* or cancer* or carcinoma* or neoplasm* or tumour* or tumor*))).ti,ab,kw. | 39998 |
| 197 | (progressive adj3 ((breast$1 or mamma or mammary) adj3 (adenocarcinoma* or cancer* or carcinoma* or neoplasm* or tumour* or tumor*))).ti,ab,kw. | 657 |
| 198 | (secondary adj3 ((breast$1 or mamma or mammary) adj3 (adenocarcinoma* or cancer* or carcinoma* or neoplasm* or tumour* or tumor*))).ti,ab,kw. | 2764 |
| 199 | (terminal adj3 ((breast$1 or mamma or mammary) adj3 (adenocarcinoma* or cancer* or carcinoma* or neoplasm* or tumour* or tumor*))).ti,ab,kw. | 174 |
| 200 | or/193-199 [METASTATIC BREAST CANCER] | 139968 |
| 201 | 176 or 192 or 200 [BREAST CANCERS OF INTEREST] | 343199 |
| 202 | exp Infant/ not exp Adult/ | 1857551 |
| 203 | exp Child/ not exp Adult/ | 3735929 |
| 204 | Adolescent/ not exp Adult/ | 1408289 |
| 205 | 201 not (202 or 203 or 204) [INFANT-, CHILD-, ADOLESCENT-ONLY REMOVED] | 342365 |
| 206 | Palbociclib.mp. | 9922 |
| 207 | (palbociclib or ibrance$2 or palbociclib or "pd 0332991" or "pd 0332991" or pd332991or pd332991 or "pf 00080665" or pf00080665).ti,ab,kw. | 6696 |
| 208 | ribociclib.mp. | 4211 |
| 209 | (ribociclib or kisqali$2 or "lee 011" or lee 011a or lee 011bba or lee 11 or lee 11a or lee 11bba or lee011 or lee011a or lee011bba or lee11 or lee11a or lee11bba).ti,ab,kw. | 2832 |
| 210 | abemaciclib.mp. | 4422 |
| 211 | (abemaciclib or bemaciclib$2 or ly 2835219 or ly2835219 or verzenio$2).ti,ab,kw. | 2892 |
| 212 | Cyclin-Dependent Kinase Inhibitor Proteins/ | 9206 |
| 213 | cyclin-dependent kinase inhibitor?.ti,ab,kw. | 15936 |
| 214 | (CDKI or CDKIs).ti,ab,kw. | 1504 |
| 215 | CKI protein?.ti,ab,kw. | 47 |
| 216 | (CIP-KIP adj2 protein?).ti,ab,kw. | 84 |
| 217 | Cyclin-Dependent Kinases/ | 22674 |
| 218 | Cyclin-Dependent Kinase 4/ | 20275 |
| 219 | Cyclin-Dependent Kinase 6/ | 11962 |
| 220 | (Cyclin-Dependent Kinase 4 or Cyclin-Dependent Kinase 6).ti,ab,kw. | 5973 |
| 221 | (CDK4* or CDK 4* or CDK6* or CDK 6*).ti,ab,kw. | 32176 |
| 222 | (Cell Division Protein Kinase 4 or PSK-J3 Kinase or PSKJ3 Kinase or p34PSK-J3 Kinase or p34PSKJ3 Kinase).ti,ab,kw. | 6 |
| 223 | (Cell Division Protein Kinase 6 or PLSTIRE Protein).ti,ab,kw. | 12 |
| 224 | or/206-223 [FIRST LINE DRUGS] | 80738 |
| 225 | 205 and 224 [BREAST CANCERS OF INTEREST - FIRST LINE DRUGS] | 11403 |
| 226 | 225 use coch,cctr [COCHRANE RECORDS] | 1221 |
| 227 | 75 or 153 or 226 [ALL DATABASES] | 3156 |
| 228 | limit 75 to dt="20231001-20241231" [Limit not valid in CCTR,CDSR,Embase; records were retained] | 135 |
| 229 | limit 153 to dc="20231001-20241231" [Limit not valid in CCTR,CDSR; records were retained] | 508 |
| 230 | 226 and (202309$ or 202310$ or 202311$ or 202312$ or 2024$).up. | 65 |
| 231 | 228 or 229 or 230 | 708 |
| 232 | remove duplicates from 231 | 578 |

EndNote deduplication process: **6** duplicates removed

# PRISMA Diagrams

## January 6, 2023 Update


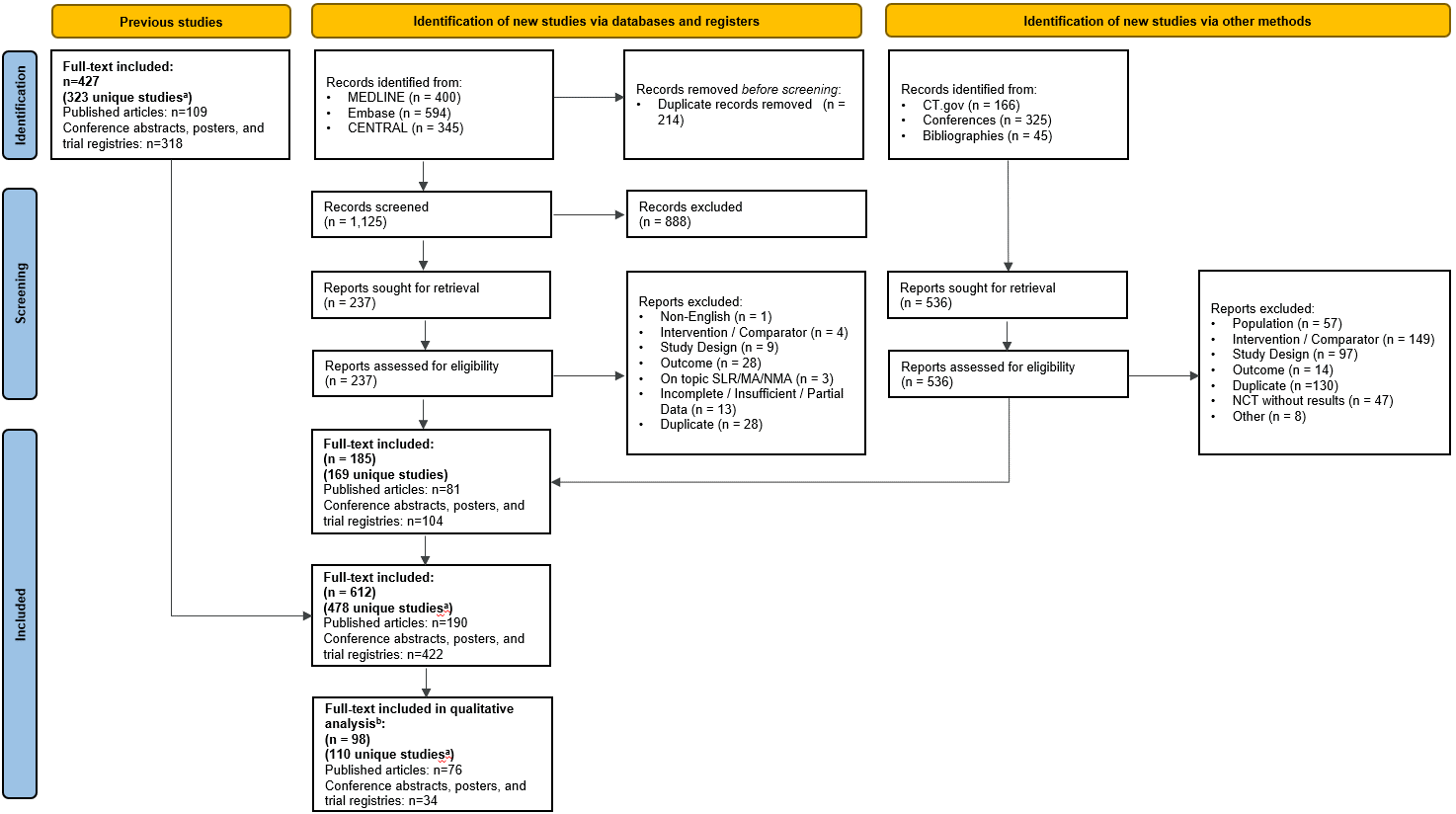


* The January 2023 update also includes records from the October 7^th^, 2020, June 1^st^, 2021, and December 1^st^, 2021 updates.

^a^ Note that references related to 14 unique studies were captured in both the previous SLRs and the December 2021 update search. Therefore, the total number of unique studies is 478 when records from all SLRs are combined.

^b^ The qualitative analysis included articles published in 2020 onwards and conference abstracts and posters published in 2022. Studies were excluded from the analysis if they had sample sizes less than 100 patients and did not specify the line of therapy.

**Abbreviations:** MA = meta-analysis; NCT = National Clinical Trial; PRISMA = Preferred Reporting Items for Systematic Reviews and Meta-Analyses; SLR = systematic literature review

From: Page MJ, McKenzie JE, Bossuyt PM, Boutron I, Hoffmann TC, Mulrow CD, et al. The PRISMA 2020 statement: an updated guideline for reporting systematic reviews. BMJ 2021;372:n71. doi: 10.1136/bmj.n71

## October 18, 2023 Update


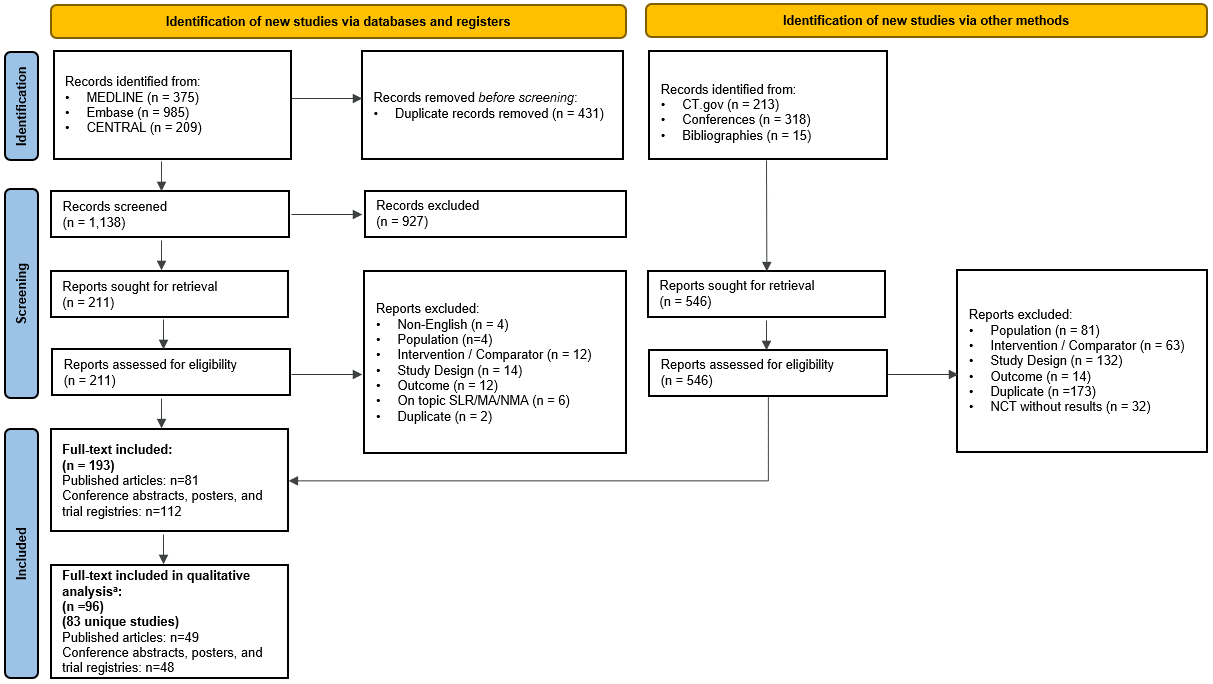


^a^ Studies were excluded from the analysis if they had sample sizes less than 100 patients and did not specify the line of therapy or type of CDK4/6i assessed.

**Abbreviations:** MA = meta-analysis; NCT = National Clinical Trial; PRISMA = Preferred Reporting Items for Systematic Reviews and Meta-Analyses; SLR = systematic literature review

From: Page MJ, McKenzie JE, Bossuyt PM, Boutron I, Hoffmann TC, Mulrow CD, et al. The PRISMA 2020 statement: an updated guideline for reporting systematic reviews. BMJ 2021;372:n71. doi: 10.1136/bmj.n71.

# List of Included Studies

| **Study Name; Reference** | **First Author** | **Year** | **Title** |
| --- | --- | --- | --- |
| PALBOSPAIN;  4203 | Anton | 2023 | Real-world treatment patterns and outcomes of patients receiving palbociclib plus endocrine therapy in Spain: Subgroup analysis based on age, sites and number of metastatic locations, menopausal status and dose received from PALBOSPAIN study |
| P-REALITY-X;  4379 | Brufsky | 2023 | Real-world treatment patterns and effectiveness of palbociclib plus an aromatase inhibitor in patients with metastatic breast cancer aged 75 years or older |
| PALOMAGE;  ASCO23-011 | Carola | 2023 | First-line systemic treatment with palbociclib in women aged ≥70 years presenting with hormone receptor-positive advanced breast cancer: Results from the PALOMAGE program. |
| RIBANNA;  ESMO23-052 | Decker | 2023 | 409P - Treatment of elderly patients (pts; > 75 years) with 1st-line ribociclib (RIB) + endocrine therapy (ET) or endocrine monotherapy in clinical routine: Real-world data from the RIBANNA study (5th interim analysis (IA)) |
| 1973 | Dennison | 2021 | Tolerability of palbociclib in younger and older patients with advanced breast cancer |
| 1809 | El Badri | 2021 | Palbociclib in combination with aromatase inhibitors in patients >= 75 years with oestrogen receptor-positive, human epidermal growth factor receptor 2 negative advanced breast cancer: A real-world multicentre UK study |
| 606 | Fountzilas | 2020 | Real-world clinical outcome and toxicity data and economic aspects in patients with advanced breast cancer treated with cyclin-dependent kinase 4/6 (CDK4/6) inhibitors combined with endocrine therapy: the experience of the Hellenic Cooperative Oncology Group. |
| 1804 | Ismail | 2021 | Palbociclib dose reductions and the effect on clinical outcomes in patients with advanced breast cancer |
| 366 | Law | 2022 | Real-World Treatment Patterns and Clinical Effectiveness of Palbociclib Plus an Aromatase Inhibitor as First-Line Therapy in Advanced/Metastatic Breast Cancer: Analysis from the US Syapse Learning Health Network |
| 271 | Mouabbi | 2022 | Histology-based survival outcomes in hormone receptor-positive metastatic breast cancer treated with targeted therapies |
| 261 | Mycock | 2022 | Real-world Treatment Patterns and Clinical Outcomes Associated With Palbociclib Combination Therapy: A Multinational, Pooled Analysis From the Ibrance Real World Insights Study |
| 3585 | Olazagasti | 2023 | A deep dive into CDK4/6 inhibitors: Evaluating real world toxicities and treatment paradigms in the elderly population |
| 204 | Pla | 2022 | Results from a real-world study of patients (pts) with hormone-receptor (HR)-positive HER2-negative metastatic breast cancer (MBC) treated with CDK4/6 inhibitors (CDK 4/6i) in three institutions |
| PERFORM;  ESMO23-012 | Radosa | 2023 | 407P - Palbociclib plus endocrine therapy in HR+/HER2- advanced breast cancer patients: Interim results of the PERFORM study |
| 3517 | Ring | 2023 | Real-World Analysis of Clinical and Demographic Characteristics, Treatment Patterns, and Outcomes in Predominantly Older Patients with HR+/HER2- Metastatic Breast Cancer Receiving Abemaciclib in Routine Clinical Practice |
| 4549 | Rugo | 2023 | Real-World Effectiveness of Palbociclib Plus Aromatase Inhibitors in African American Patients With Metastatic Breast Cancer |
| 3494 | Rugo | 2023 | Real-world comparative effectiveness of palbociclib plus letrozole versus letrozole in older patients with metastatic breast cancer |
| 1773 | Sampedro | 2021 | Observational real world data with palbociclib associated to hormone therapy for advanced breast carcinoma |
| 1198 | Schreier | 2022 | Racial disparities in neutrophil counts among patients with metastatic breast cancer during treatment with CDK4/6 inhibitors |
| REACHAUT;  SABCS23-117 | Singer | 2023 | Real-world outcomes with first-line ribociclib + endocrine therapy in patients with metastatic HR+, HER2– breast cancer: Fourth interim analysis of REACH AUT trial |
| 4506 | Tang | 2023 | Clinical Impact of CDK4/6 Inhibitors in De Novo or PR- or Very Elderly Post-Menopausal ER+/HER2- Advanced Breast Cancers |
| 3309 | Wu | 2023 | Effectiveness and Safety of Palbociclib Plus Endocrine Therapy in Patients with Advanced Breast Cancer: A Multi-Center Study in China |
| 1624 | Zhang | 2021 | The efficacy and safety of palbociclib combined with endocrine therapy in patients with hormone receptor-positive HER2-negative advanced breast cancer: a multi-center retrospective analysis |

# Quality Assessment
